# Synthesis of Indoloquinolines: An Intramolecular Cyclization Leading to Advanced Perophoramidine-Relevant Intermediates

**DOI:** 10.3390/molecules26196039

**Published:** 2021-10-05

**Authors:** Craig A. Johnston, David B. Cordes, Tomas Lebl, Alexandra M. Z. Slawin, Nicholas J. Westwood

**Affiliations:** School of Chemistry and Biomedical Sciences Research Complex, University of St. Andrews and EaStCHEM, North Haugh, St. Andrews KY16 9ST, UK; craig.johnston85@gmail.com (C.A.J.); dbc21@st-andrews.ac.uk (D.B.C.); tl12@st-andrews.ac.uk (T.L.); amzs@st-andrews.ac.uk (A.M.Z.S.)

**Keywords:** perophoramidine, natural product, Claisen rearrangement, indoloquinoline, intramolecular cyclization, X-ray structure

## Abstract

The bioactive natural product perophoramidine has proved a challenging synthetic target. An alternative route to its indolo[2,3-b]quinolone core structure involving a N-chlorosuccinimde-mediated intramolecular cyclization reaction is reported. Attempts to progress towards the natural product are also discussed with an unexpected deep-seated rearrangement of the core structure occurring during an attempted iodoetherification reaction. X-ray crystallographic analysis provides important analytical confirmation of assigned structures.

## 1. Introduction

Attempts to prepare complex bioactive natural products often test synthetic methodology under very challenging circumstances. In addition, unexpected outcomes, in what initially look like simple transformations, frequently occur and provide interesting analytical conundrums. One approach to solving these structural questions is the use of small-molecule X-ray crystallography which continues to play an essential role in developing routes to complex molecules. Through collaborations, such as ours with Professor Alexandra Slawin, difficult analytical challenges are frequently solved with apparent ease. For anyone who has had the pleasure to work with someone with Professor Slawin’s level of expertise, the phrase “Oh of course Alex, now you’ve pointed it out that has to be the structure” will probably be familiar!

**Scheme 1:**
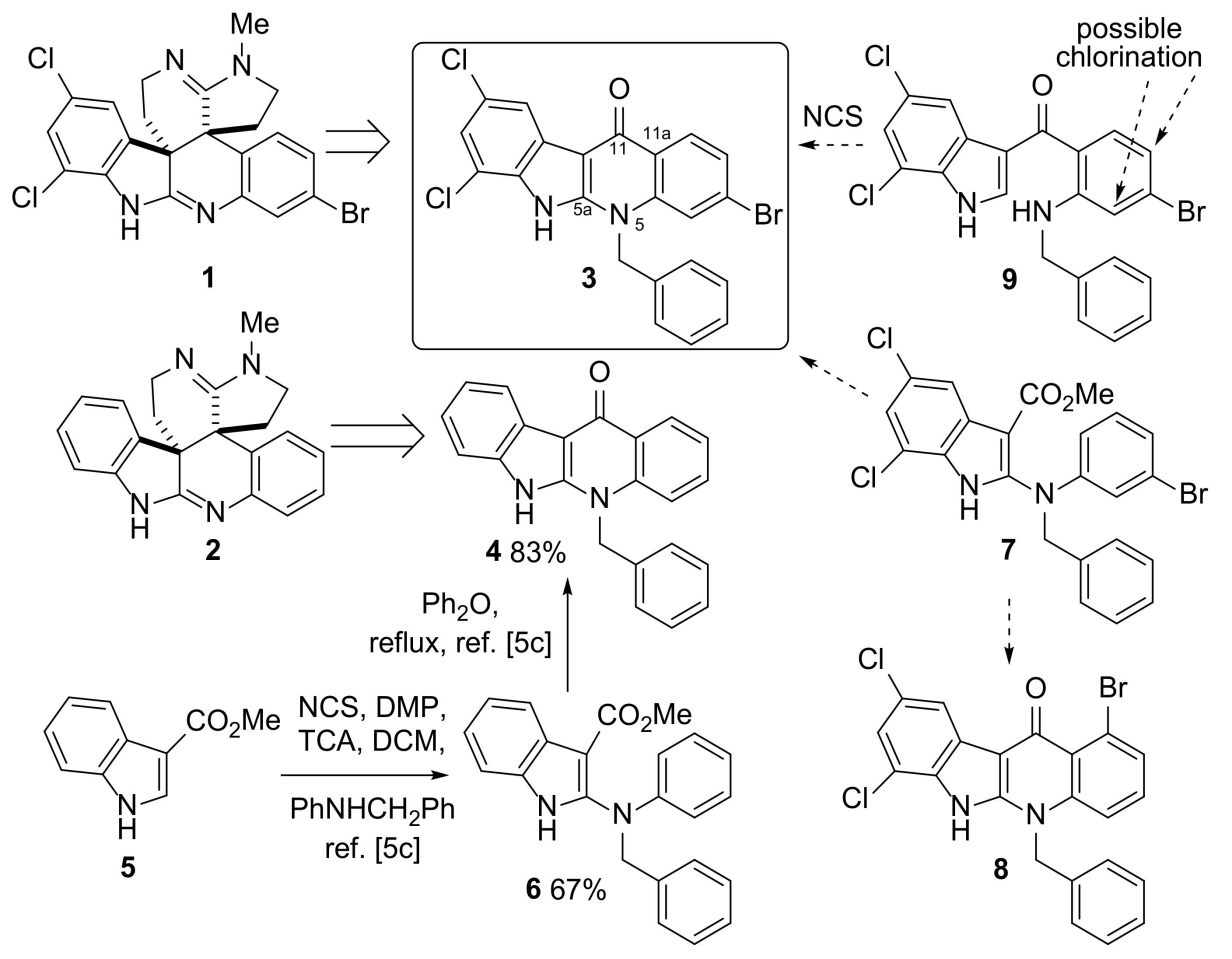
Structures of the natural product perophoramidine **1** and the product prepared by Ireland et al. [1] following dehalogenation, dehaloperophoramidine **2**. Previous reports [5c] described the synthesis of **4** via a NCS-mediated coupling reaction of **5** and subsequent cyclization of **6**. **4** was then converted to **2** [21]. In this work, access to the analogous intermediate **3** was explored with two possible routes either via **7** or an intramolecular NCS-mediated cyclization of **9** considered. An alternative product, compound **8**, from a potential cyclization reaction of **7** was possible. Chlorination of the aniline ring in **9** was also a potential side reaction. NCS = N-chlorosuccinimide; DMP = N,N’-dimethylpiperazine; TCA = trichloroacetic acid.

The structure of the natural product perophoramidine **1** (Scheme 1) was first reported in 2002 by Ireland [1] although earlier biosynthetic proposals, synthetic and small molecule X-ray crystallographic work had sparked interest in closely related alkaloids [2,3,4,5,6,7,8,9,10]. To date, a number of elegant total syntheses of perophoramidine **1** have been reported [11,12,13,14,15,16] along with a range of other attempts [17,18]. In addition, in their initial report, Ireland et al. reported the dehalogenation of perophoramidine **1** under hydrogenation conditions using HCO_2_NH_4_ and Pd/C in MeOH. This led to the formation of a compound that they named dehaloperophoramidine **2** (Scheme 1) [1]. Additional syntheses of compound **2** have been reported [19,20,21,22], with one of these routes to **2** being developed in our laboratory [21]. As our studies to **2** progressed, investigations into the synthesis of the halogen-containing **1** were also carried out.

Here, we report the successful synthesis of the halogen-containing analogue **3** of compound **4**, which was a key intermediate in our synthesis of **2** (Scheme 1) [21]. Attempts to progress forward from **3** towards **1**, using an alternative route to that reported by us for the synthesis of **2** [21], are also described. In addition, we include the structural assignment of two of the prepared compounds by small-molecule X-ray crystallographic analysis, bringing to 18 the total number of times this technique has guided this overall program of work (for the previous small molecule X-ray crystallographic structures see CCDC 737646-737648, 1486344, 1478152-4, 1582892-7 and 1811882-4).

## 2. Results

### 2.1. Proposed Route to Halogenated Intermediate ***3***

We have previously reported [21] a robust and scalable route to the indolo[2,3-*b*]quinoline core structure of **4** using a *N*-chlorosuccinimide (NCS)-mediated coupling of methyl indole-3-carboxylate (**5**) with *N*-benzylaniline followed by cyclization of the product **6** in refluxing diphenyl ether (Scheme 1) [23]. This reaction sequence proved a suitable starting point for the synthesis of dehaloperophoramidine **2** [21]. Whilst this approach was relatively straightforward and high yielding in the case of **4**, it seemed likely that the analogous reaction using substrate **7** with the required halogenation pattern to prepare perophoramidine **1** would lead to the formation of regioisomers **3** and **8** (Scheme 1). It was envisaged that separation of **3** and **8** would prove challenging as these type of compounds exhibit low solubility in organic solvents.

**Scheme 2:**
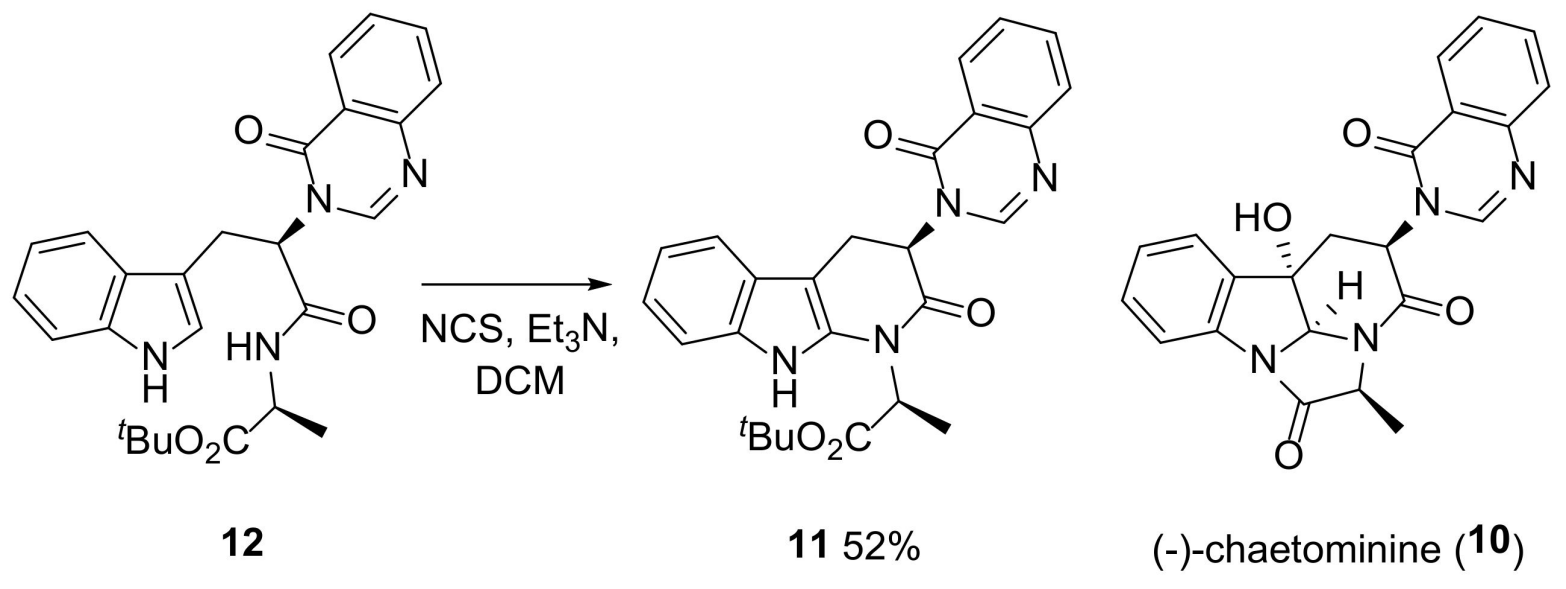
Reported synthesis of **11** via a NCS-mediated formation of the C–N bond at the indole 2-position during the preparation of the natural product (–)-chaetominine (**10**) [24].

To avoid this issue, an alternative approach was proposed involving a NCS-mediated cyclization reaction of substituted indole **9** to form the N5–C5a bond in **3** after the C11–C11a bond. This is an intramolecular version of the reaction used to form **6** (Scheme 1). NCS-mediated intramolecular cyclization reactions have recently been used to form a C–N bond at the indole 2-position in the total synthesis of (−)-chaetominine (**10**) [8] with tetrahydro-1*H*-pyrido[2,3-*b*]indole (**11**) being prepared from substituted indole **12** using NCS (1.3 equiv.) and Et_3_N (4 equiv.) in DCM in 52% yield (Scheme 2). One possible challenge with this approach in our system was competing chlorination in the aniline ring in **9** (Scheme 1). Given difficulties in predicting the result of this competition a priori, it was decided to investigate this proposed route to **3**.

### 2.2. Synthesis of Halogenated Intermediate ***3***

**Scheme 3:**
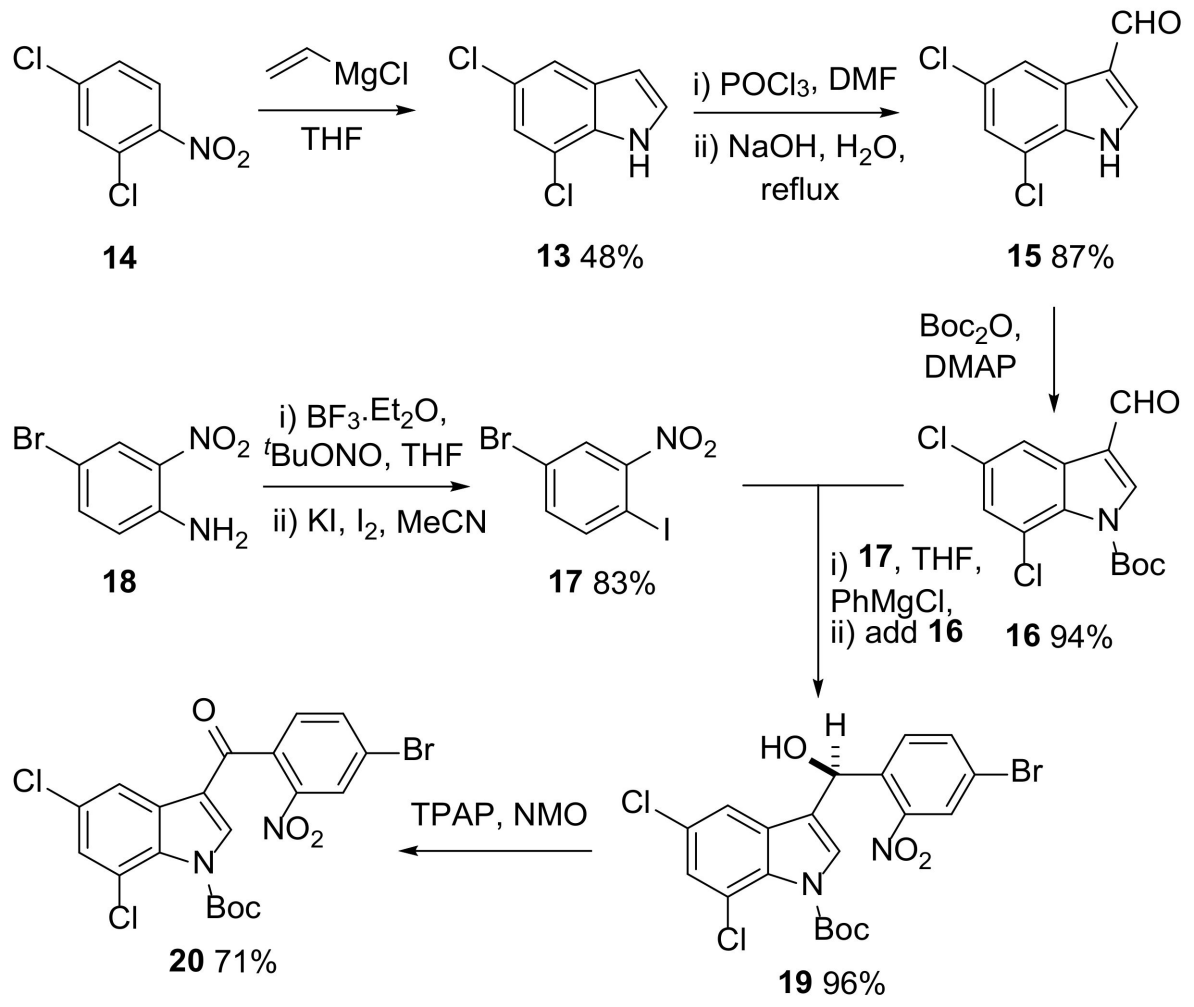
Synthesis of ketone **20** involving generation of indole **13** using a Bartoli reaction [27,28] and a Knochel-type coupling reaction. TPAP = tetrapropylammonium perruthenate; NMO = N-methylmorpholine N-oxide.

Whilst several methods are available for the synthesis of 5,7-dichloroindole (**13**) [24,25,26,27,28] including electrochemical-mediated cyclization [25] and gold-catalyzed annulation of the corresponding 2-alkynylaniline [26], it was decided to use the Bartoli indole synthesis [27,28] as multi-gram quantities of **13** were required (Scheme 3). Reaction of 2,4-dichloronitrobenzene (**14**) with vinylmagnesium chloride [28] gave indole **13** with key steps in the process involving a [3,3]-sigmatropic rearrangement followed by cyclization onto the resulting aldehyde to form the 5-membered ring. The yield of this reaction was relatively low (48%, in line with the literature precedent [28]); however, the starting materials were readily available and the reaction could be carried out to give almost 9 g of **13**. Indole **13** was subsequently converted to aldehyde **15** using a Vilsmeier-Haack reaction [29] and Boc protection of the indole nitrogen using di-*tert*-butyl dicarbonate (Boc_2_O) in the presence of 4-dimethylaminopyridine (DMAP) formed **16** in high yield (Scheme 3).

Aryl iodide **17** was then synthesized from **18** in 83% yield using a literature procedure (Scheme 3) [30]. Having prepared intermediates **16** and **17**, a Grignard-mediated coupling reaction was attempted. This reaction was based on a method reported by Knochel et al. in which aryl rings containing an iodine atom *ortho* to a nitro group can undergo I-Mg exchange when treated with phenylmagnesium chloride [31,32]. The resulting Grignard reagent can then react with an electrophile to form a new carbon–carbon bond. Interestingly, more reactive Grignard reagents such as methyl magnesium chloride react with the nitro group, leading to complex mixtures of products. The reaction is also reported to be unsuccessful when *meta-* or *para*-iodonitrobenzenes are used [31,32], leading to the proposal that chelation of the nitro group to the magnesium atom stabilizes the *ortho*-substituted Grignard reagent. The treatment of iodide **17** with phenylmagnesium chloride at −40 °C for 1 h followed by reaction with aldehyde **16** gave alcohol **19** (racemate, Scheme 3) in excellent yield, even on a multiple-gram scale. Alcohol **19** was then oxidized to ketone **20** using tetrapropylammonium perruthenate (TPAP, 5 mol%) and co-oxidant *N*-methylmorpholine *N*-oxide (NMO) (Scheme 3) [33].

**Scheme 4:**
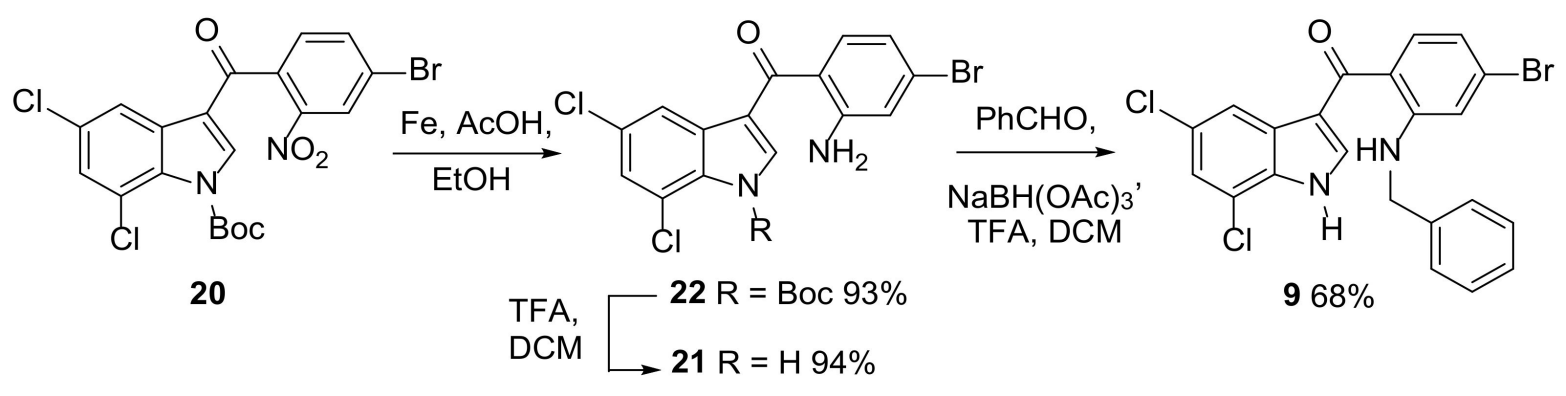
Nitro group reduction and reductive amination.

Attempts were made to reduce the nitro group to the corresponding amine and remove the Boc protecting group in one step by refluxing **20** in acetic acid and ethanol in the presence of iron powder. However, this reaction produced a mixture of the expected product **21** and Boc protected **22**. A two step protocol using iron in acetic acid and ethanol followed by treatment of purified **22** with TFA in DCM gave **21** in an excellent yield over the two steps (Scheme 4). The required reductive N-benzylation of **21** proved more challenging. When **21** was heated with benzaldehyde in refluxing toluene for six hours followed by the addition of sodium triacetoxyborohydride (STAB) the required product **9** was obtained in only moderate yield presumably due to the relatively poor nucleophilicity of the aniline nitrogen in **21** disfavouring initial imine formation. An alternative improved procedure based on the report of Boros et al. [34] was eventually found. This involved the additional use of TFA and so enabled telescoping of the conversion of **22** to **9** which after optimization of this reaction (a second aliquot of the STAB/TFA solution after 30 min) enabled the formation of **9** from **22** to 68% with a small quantity of **21** also being obtained (Scheme 4).

**Scheme 5:**
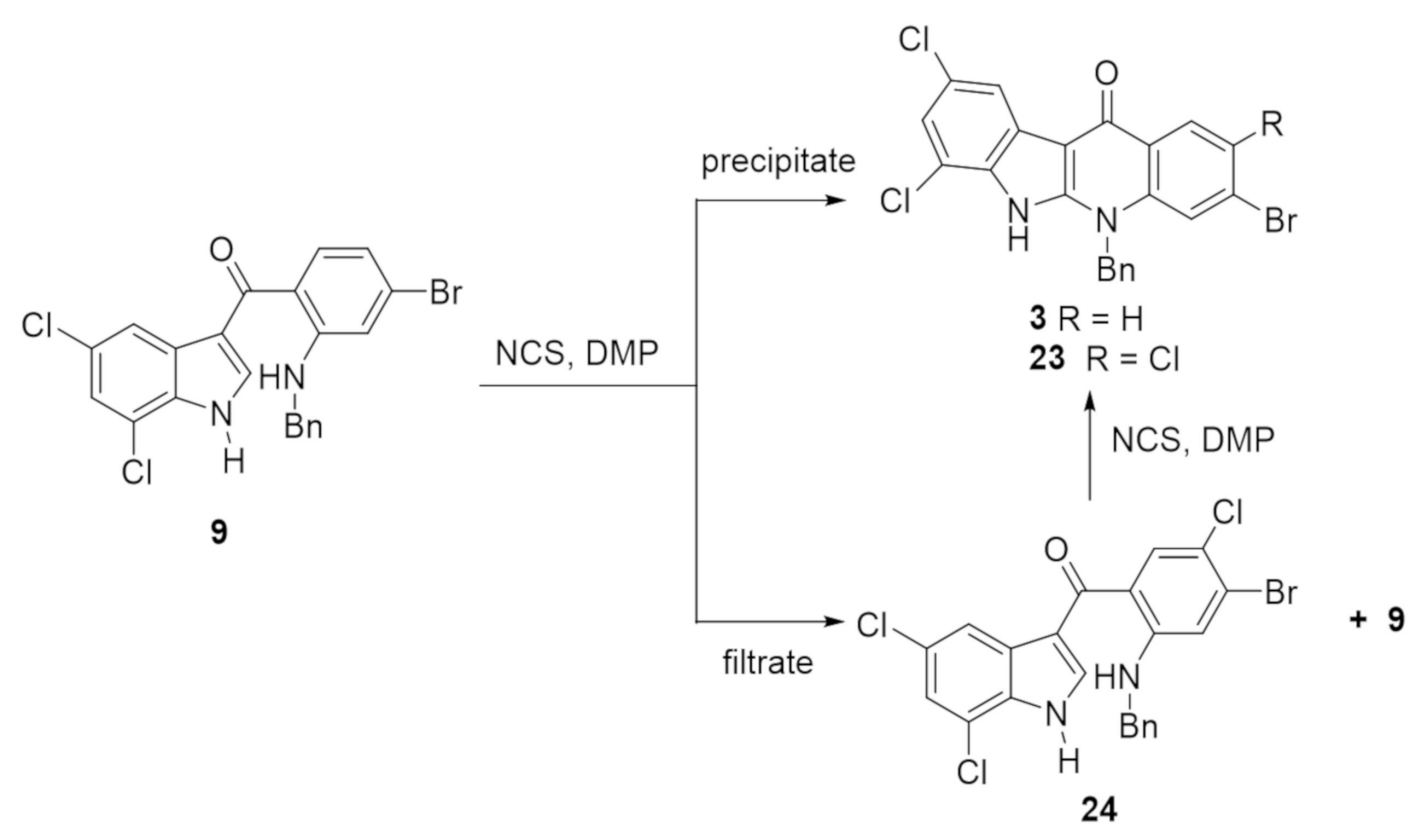
Preliminary attempts at NCS-mediated cyclization of **9** using NCS (2.0 equiv.), DMP (0.56 equiv.) in DCM at room temperature for 28 h led to formation of a precipitate that contained the desired cyclized product **3** and a second product assigned as **23** (Figure S3, see Supplementary Material for analytical data for **23** and **24**). **23** was independently prepared from an isolated sample of **24** (NCS (1.0 equiv.), DMP (0.56 equiv.) in DCM at room temperature for 16 h). Significant optimization of this reaction was required.

After carrying out a series of model studies (see Supplementary Material), a NCS-mediated cyclization of **9** was attempted. The initial conditions used were NCS (2.0 equiv.) and DMP (0.56 equiv.) in DCM at room temperature overnight. After stirring for 28 h, it was observed that a small amount of precipitate was formed. The precipitate was isolated by filtration, washed with DCM and analyzed by ^1^H NMR in d6-DMSO (Figure S2). The NMR and mass spectrometric analysis was consistent with the presence of two compounds with m/z values equal to the required product **3** and a chlorinated analogue of **3** (assigned structure **23** (Scheme 5 and Figure S3), vide infra). Analysis of the filtrate and washings from the reaction indicated that again two main compounds were present, one of which was unreacted **9**. The second compound was assigned as **24** (Scheme 5), which was presumably formed by NCS-mediated chlorination at the aniline 4-position rather than at the indole 3-position of **9**. The isolation of **24** also led to the proposal that the unidentified chlorinated analogue of **3** present in the precipitate was in fact **23**, the cyclized version of **24**. To confirm this, a small-scale reaction of **24** with NCS and base was carried out, giving **23** (Scheme 5, Figure S3 and Supplementary Material for protocols and analytical data for **23** and **24**).

**Scheme 6:**
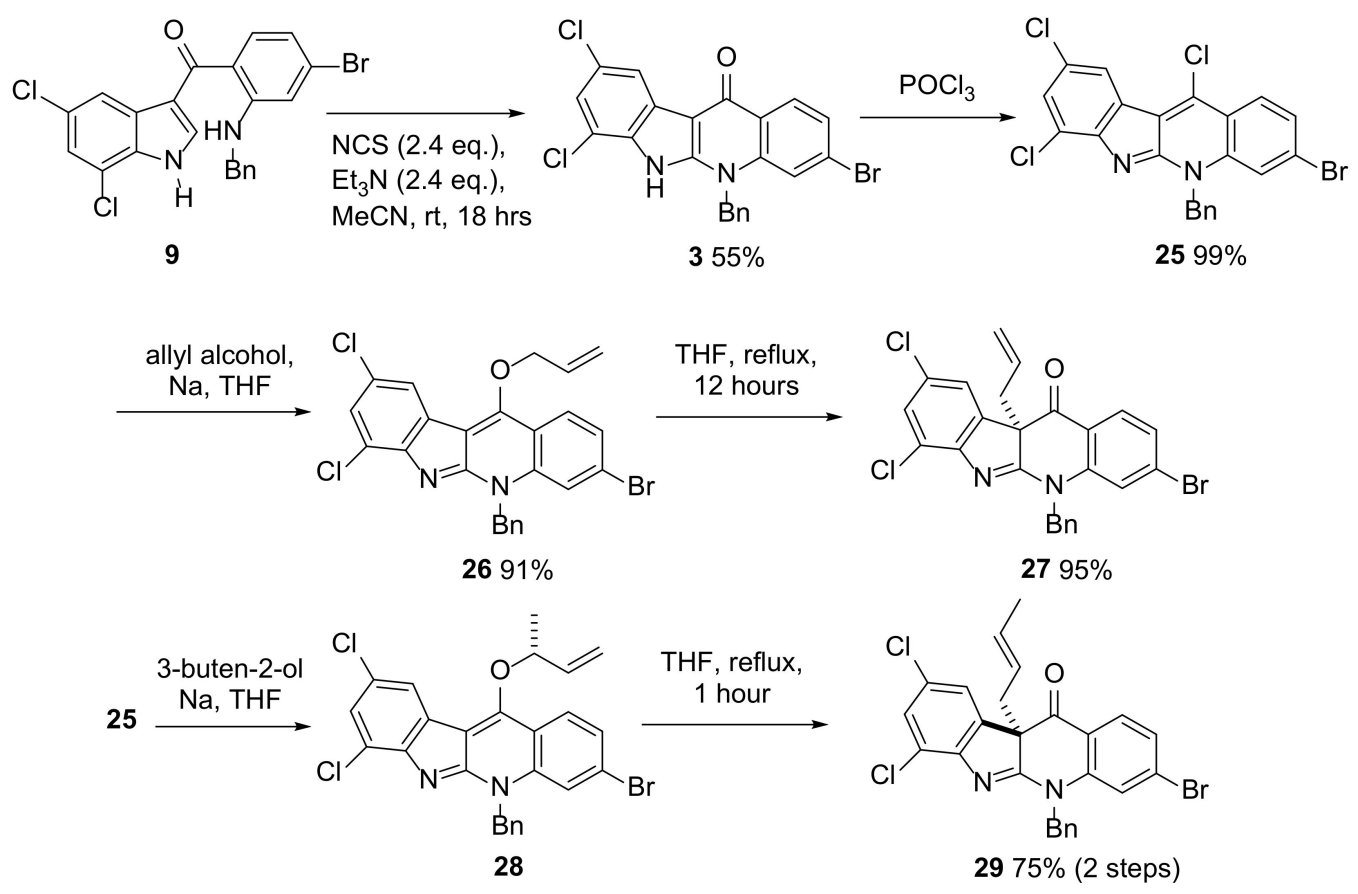
Optimized conditions for the conversion of **9** to **3** and instalment of an all-carbon quaternary center in compounds **27** and **29**. Insights into the impact of the halogen substituents on both the addition-elimination and Claisen rearrangement steps were gained.

Optimization of this reaction started with a solvent screen based on literature precedent [23,24,35,36]. The reaction of **9** with NCS (1.0 equiv.) and DMP (0.56 equiv.) was carried out in seven common solvents (DCM, MeOH, acetone, THF, CH_3_CN, hexane and toluene) and the amount of precipitate isolated and the ratio of **9**:**24** in the filtrates was determined (Table S1). Acetonitrile was judged as the preferred solvent as the largest amount of precipitate was formed (cyclized products, Table S1, Entry 5). As related reactions have been reported using a range of bases [23,35,36,37,38,39,40], it was decided to react **9** with NCS (1.2 equiv.) in acetonitrile using six different bases (DMP, NaH, Et_3_N, DMAP, DIPEA and pyridine, Table S2). In brief, the use of Et_3_N gave the highest product yield (48%) with only the required **3** being present in the precipitate and starting material **9** being the dominant product in the filtrate (Table S2, Entry 3). Further optimization of this reaction found that when 2.4 equivalents of both NCS and Et_3_N were used at room temperature for 18 h, **3** could be isolated in 55% yield with **9** (21%) recovered on purification by column chromatography of the filtrate (Scheme 6 and Supplementary Material demonstrating the reproducibility of this reaction up to a 2 g scale).

### 2.3. Installation of the First All-Carbon Quaternary Center and X-ray Structure Determination of Advanced Intermediate ***27***

**Figure 1:**
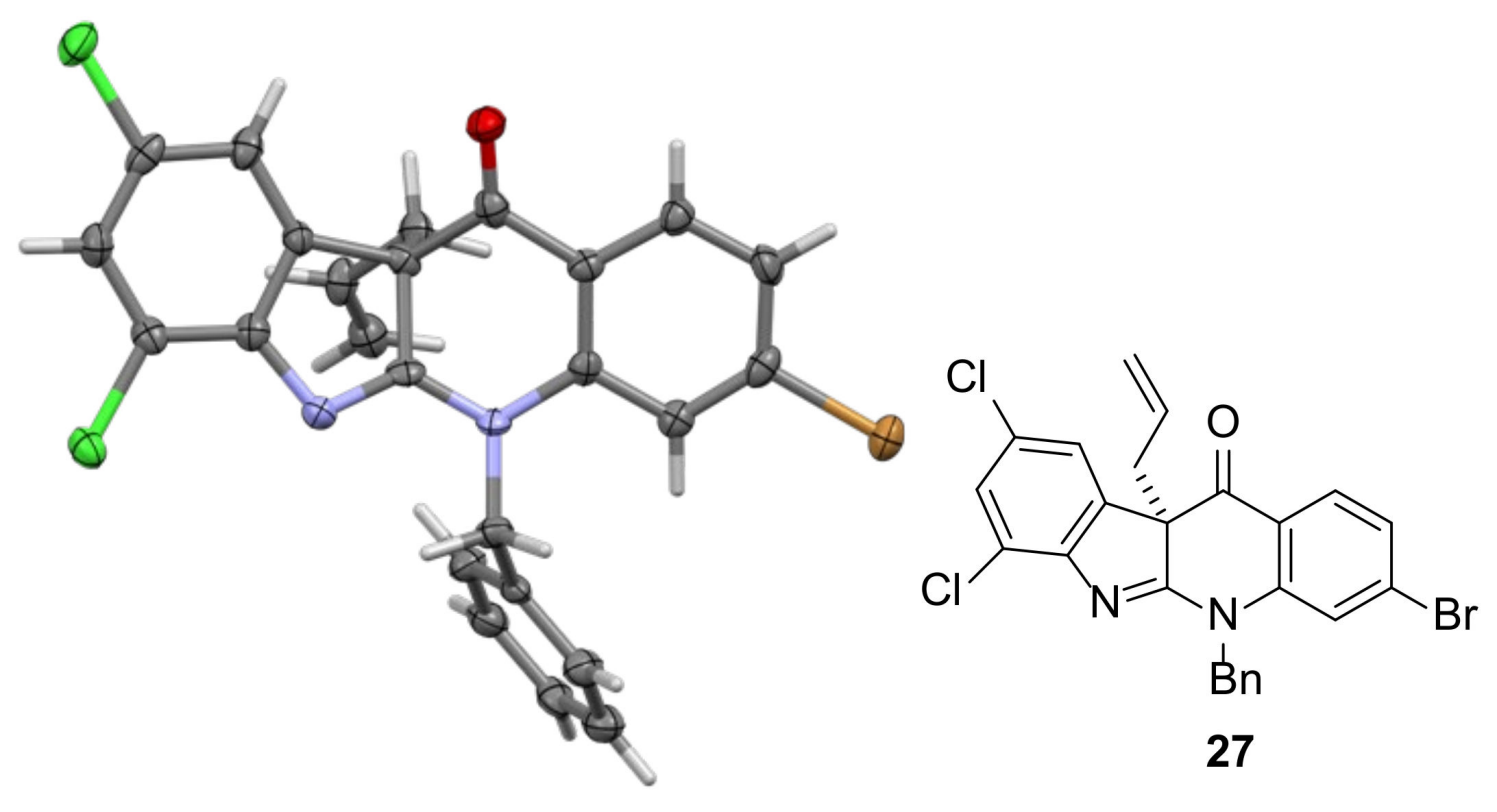
A view of the X-ray crystal structure of **27** (ellipsoids drawn at the 50 % probability level) used to confirm its structure.

After developing the route to ketone **3**, the instalment of the first of the all-carbon quaternary centers (Scheme 1 and Scheme 6) present in perophoramidine **1** was achieved over 3 steps. Reaction of **3** with POCl_3_ gave **25** in almost quantitative yield. Substitution of one of the chlorines in **25** using sodium allyloxide gave allyl ether **26** (Scheme 6) which underwent Claisen rearrangement to form ketone **27** on heating at reflux in THF for 12 h. Recrystallization of **27** provided crystals suitable for X-ray crystallographic analysis, which enabled confirmation of its structure (Figure 1). This three-step sequence was also successful when the alkoxide formed from the reaction of racemic 3-buten-2-ol with sodium was used. As observed previously in the dehalo system (Scheme S4) [21], the Claisen rearrangement of the crotyl-containing **28** generated from **25** was feasible at room temperature (unlike allyl-containing **26** which does not rearrange at all at room temperature) and as a result it was not possible to isolate **28** in pure form. Heating the obtained sample of predominantly **28** at reflux in THF only required 1 h for full conversion to **29** in 75% yield over the two steps (*c.f.* the 12 h at reflux in THF required for complete conversion of **26** to **27** and the 5 h required in the analogous reaction in the crotyl-dehalo series, Scheme S4). It is clear that the presence of the halogens in **28** accelerated the Claisen rearrangement. In this sequence it was also interesting to note that the alkoxide addition-elimination reactions of **25** to give **26** or **28** proceeded much faster than the corresponding reaction of dehalo analogue which required 18 h to convert fully [23]. This is likely due to the electron-withdrawing effect of the halogen substituents in **25** promoting nucleophilic addition of the alkoxide. Although the synthesis of intermediate **29** was carried out in such a way that racemic product was obtained, the use of enantiomerically enriched (*R*)-3-buten-2-ol would enable the formation of the C-10b quaternary center with the correct absolute stereochemistry required for an asymmetric synthesis of perophoramidine **1** [23].

### 2.4. Attempted Progress towards Perophoramidine ***1***

**Scheme 7:**
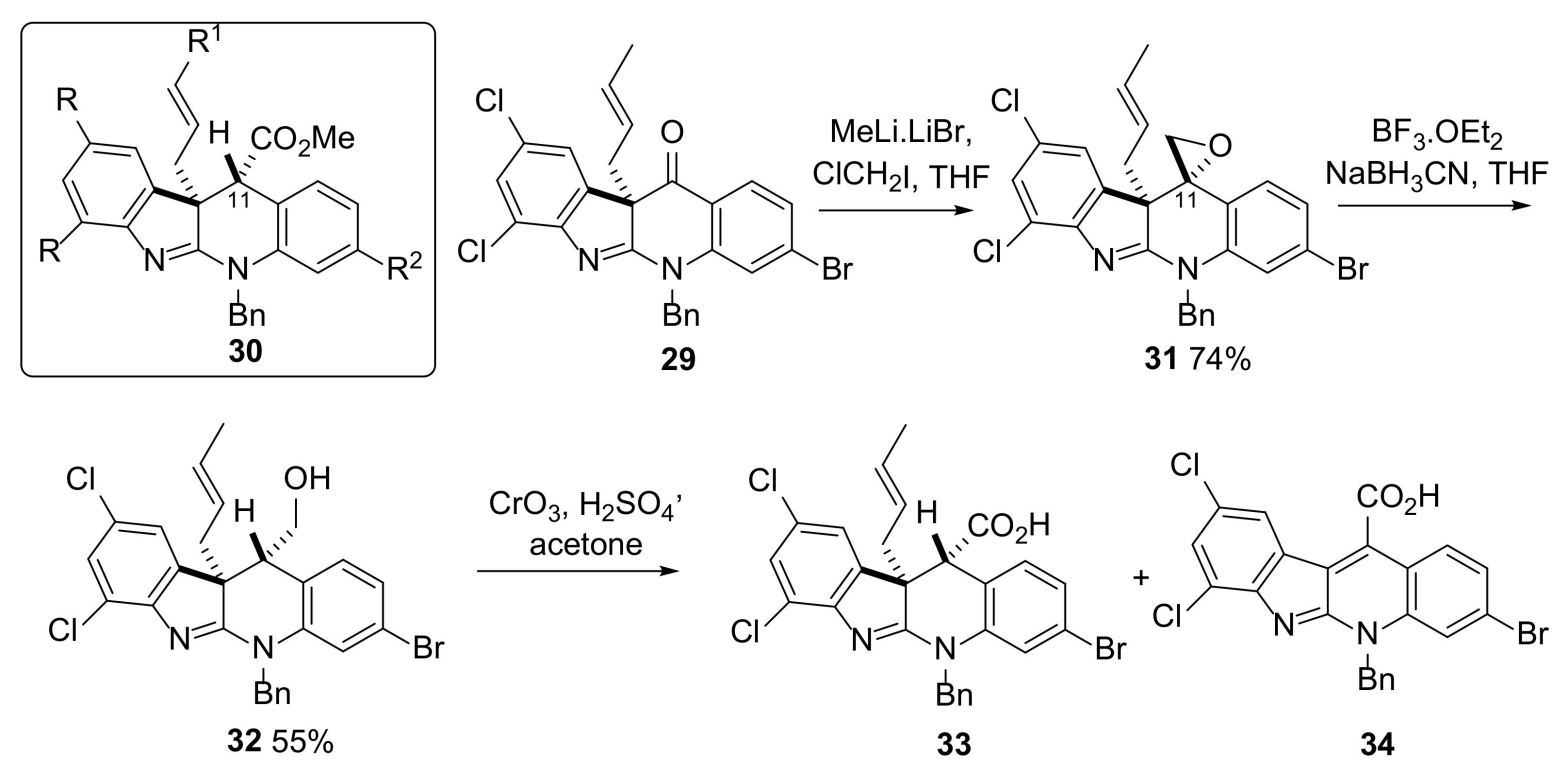
Attempted formation of ester **30** (R = Cl, R^1^ = Me, R^2^ = Br) to enable ester alkylation as a method of incorporating the second all-carbon quaternary center. Reaction of **32** under Jones oxidation conditions gave **34** rather than the desired **33**.

The second half of this report briefly describes two approaches that were attempted to progress from advanced synthetic intermediates **27** and **29** towards perophoramidine **1**. Whilst ultimately unsuccessful, these approaches did provide additional insights into the inherent reactivity of this complex system. One of the initial strategies employed by others [17,41] and us [42] for the synthesis of **1** and **2** involved the preparation of a C11-ester-containing intermediate, in our case, of general structure **30** (Scheme 7and Scheme S5). The approach attempted here for installing a C11-ester involved reaction of the previously prepared **29** with in situ-generated chloromethyl lithium to form epoxide **31** in good yield (Scheme 7). The relative stereochemistry of **31** was tentatively assigned based on the absence of a nOe correlation between the CH_2_ of the epoxide and any of the protons of the crotyl chain. In addition, the assigned stereochemistry in **31** was expected due to the attack of the chloromethyl lithium from the least hindered face of the molecule as was observed in previous studies in which proof of structure had been obtained through small-molecule X-crystal structure analysis of the analogous dehalo-epoxide [21]. Reductive opening of **31** to give **32** was then achieved using excess boron trifluoride and sodium cyanoborohydride in moderate yield. It was believed that this reaction proceeded with inversion of configuration at the C-11 stereocenter due to hydride attack occurring from the least hindered face, again consistent with previously reported studies in which small-molecule X-ray analysis of a related dehalo-analogue had been achieved [42]. Unfortunately, the results obtained from the Jones oxidation [43] of **32** were different to those of the previously reported non-halogenated alcohols [42]. Following the reaction by LC–MS showed that after just 5 min reaction time, **32** was almost completely consumed with a number of different product peaks being observed in the LC–MS spectrum. From the complex mixture of products obtained at this time point, evidence for the presence of **33** was obtained. After an increased reaction time (5 h), the initially complex mixture simplified with the major product being assigned as the decomposition product **34** (Scheme 7 and Figure S4, observed *m/z* = 501.17 [M+H]^+^; theoretical m/z for formation of **34**, C_23_H_13_^81^Br^35^Cl_2_N_2_O_2_ [M+H]^+^ 500.96). No evidence for the presence of **33** in the reaction mixture at the 5 h time point was found.

**Scheme 8:**
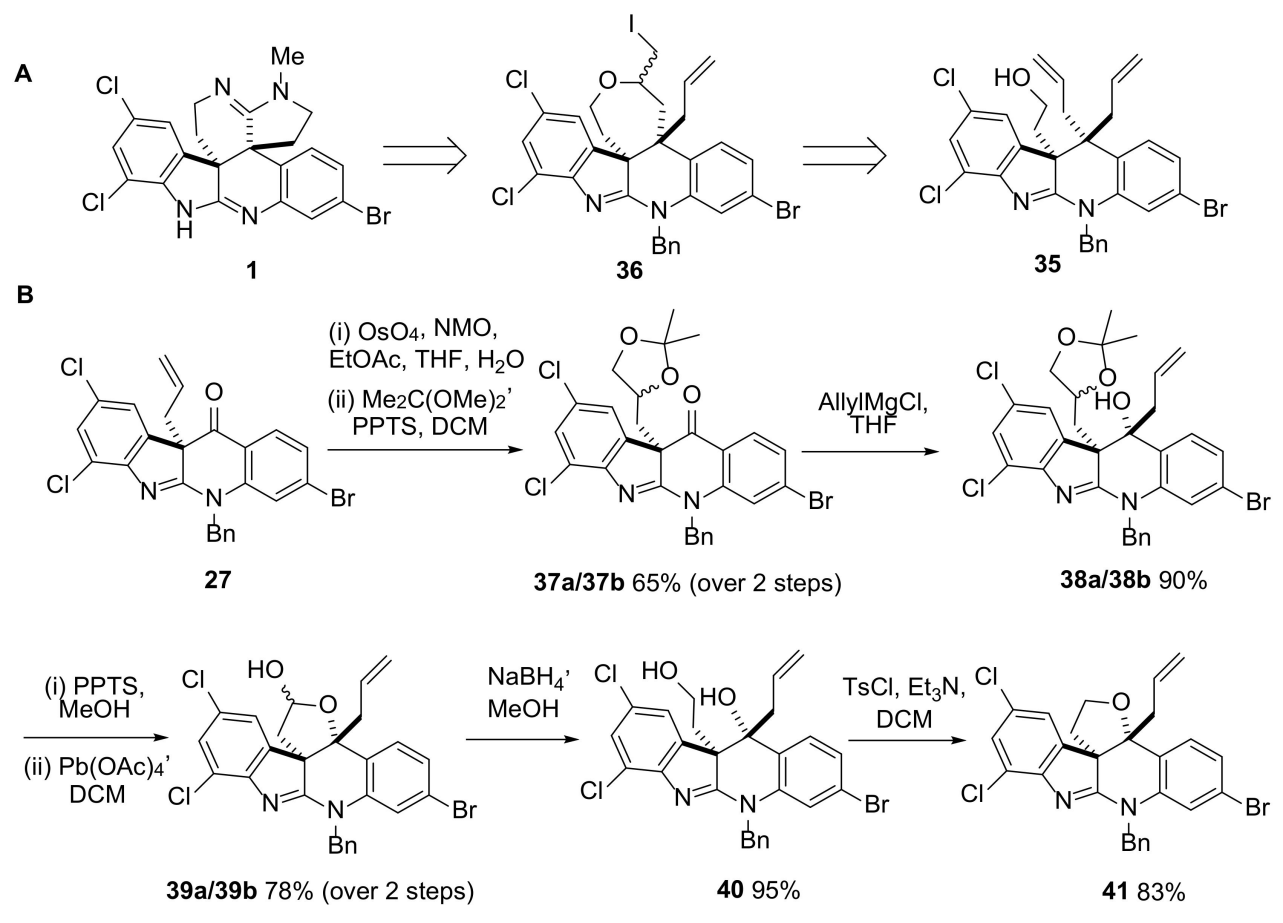
**A.** One possible approach to perophoramidine **1** via diallyl-containing compound **35**. Differentiation between the two allyl groups in **35** could potentially be achieved through an iodoetherification protocol. **B.** Synthesis of cyclic ether **41**, a precursor to **35**. PPTS = pyridinium p-toluenesulfonate, TsCl = p-toluenesulfonyl chloride.

As conversion of **32** to **34** clearly involved loss of the crotyl group (for one possible mechanism see Scheme S6), it was next decided to develop the crotyl chain in **29** (or in fact the allyl chain in **27**, Scheme 8) by reacting the double bond. Building on an approach previously reported by us in the dehalo series [44], **35** became the new target molecule. Subsequent conversion of **35** to **36** could provide an approach to perophoramidine **1** by differentiating between the two allyl groups as seen in a related system [21].

**Figure 2:**
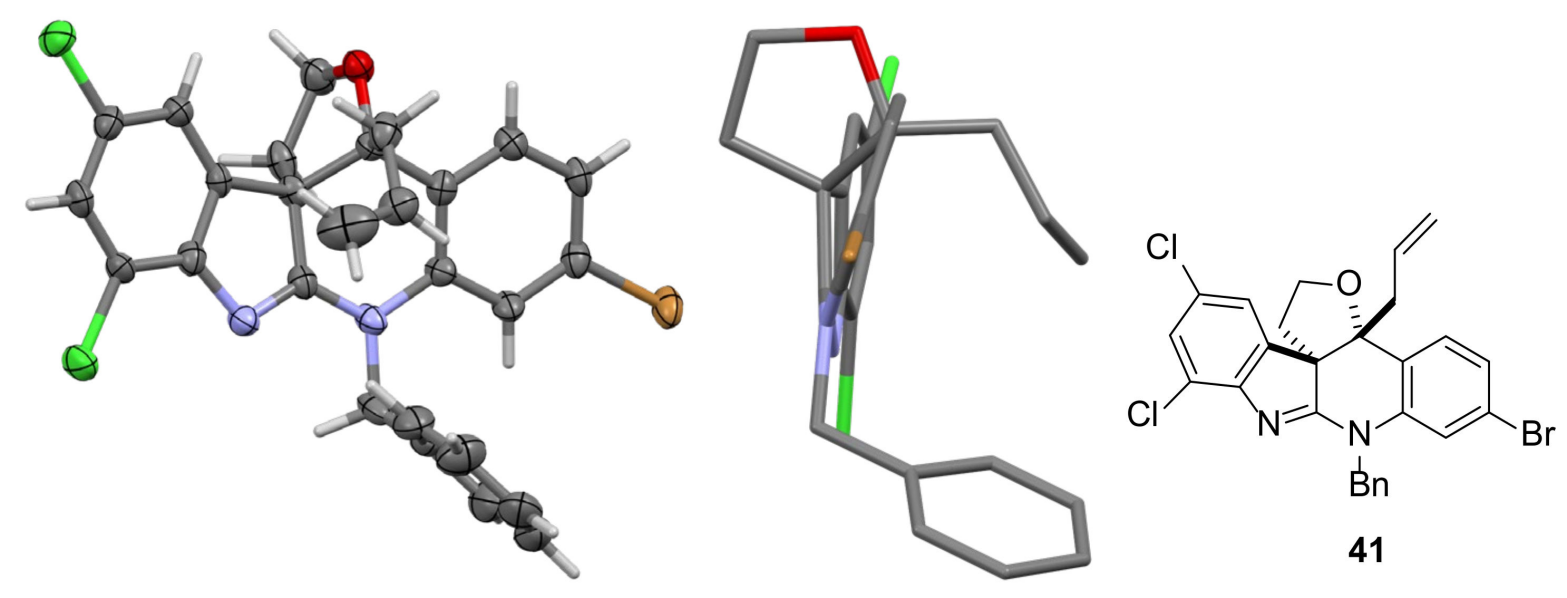
Two views of the X-ray crystal structure of **41** (ellipsoids drawn at the 50 % probability level) used to confirm its structure.

After dihydroxylation of the allyl group in **27**, the corresponding diastereomeric acetonides **37a**/**37b** (1:1 mixture) were formed in 65% yield over the two steps. Reaction of **37a**/**37b** with allylmagnesium chloride proceeded in high yield to give only two of the possible diastereomers (assigned structures as shown in **38a**/**38b** based on expected stereochemical outcome of addition to the ketone). Deprotection of the acetonide group in **38a**/**38b** was achieved under relatively mild conditions using PPTS in methanol [45] and the resulting crude reaction mixture was treated with lead tetraacetate, leading to lactols **39a**/**39b** still as a 1:1 mixture of diastereomers. Reduction of **39a**/**39b** with sodium borohydride in methanol gave **40** which could be converted to cyclic ether **41** in excellent yield over the two steps. Importantly, after dealing with a series of diastereomeric mixtures for several steps in this sequence, recrystallization of **41** by slow evaporation of a solution of **41** in ethyl acetate provided crystals suitable for small-molecule X-ray crystallography. From the data obtained, it was clear that the stereochemistry at the two stereogenic centers in **41** was as planned. This places the ether ring almost perpendicular to the tetracyclic core of **41**, with the allyl substituent on the opposite face to the ether ring (Figure 2).

**Scheme 9:**
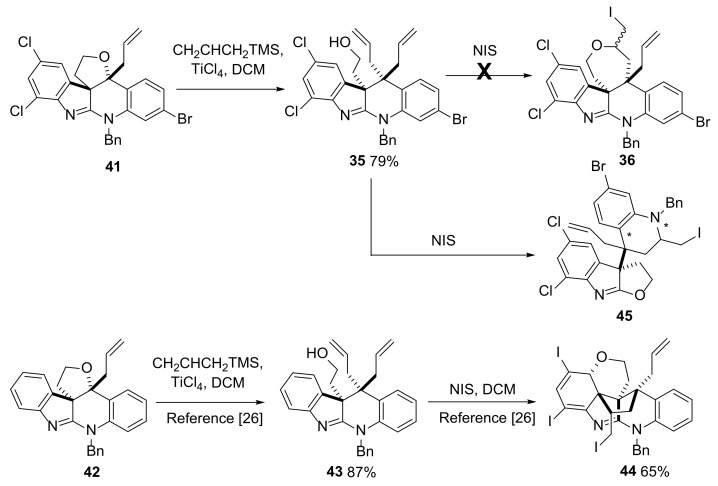
Attempted conversion of **35** to **36** led instead to formation of **45**.

Opening of the cyclic ether in **41** proceeded smoothly with **35** being obtained in 79% yield after reaction with TiCl_4_ and allyltrimethylsilane in DCM at −78 °C for 4.5 h. The presence of the halogen substituents in **35** had little effect on this robust reaction which has previously been reported to proceed in a similar manner with the non-halogenated analogue **42** to give **43** (87% yield [44]). Previous attempts to differentiate between the two allyl groups present in **43** using an iodoetherification reaction resulted in the formation of **44**, the structural assignment of which (especially the relative stereochemistry) relied heavily on X-ray crystallographic analysis [44]. It was proposed that the presence of the chlorine substitutents in **35** may steer the outcome of this reaction away from that observed with **43** as a reduction in the electron density in the aryl ring due to the presence of the net deactivating chlorines would be expected to slow the proposed reaction of this ring with an intermediate iodonium ion central to the formation of **44** from **43** (Scheme 9 and Scheme S7).

Reaction of **35** with excess NIS in freshly base-washed CDCl_3_ was followed by purification of the crude reaction mixture by column chromatography using triethylamine treated silica. Mass spectrometric analysis of the product indicated that one iodine atom had been added to the molecule, possibly consistent with the desired formation of **36**. However, detailed NMR analysis indicated that the structure of the major product from this reaction was not the planned iodoether **36** or an analogue of **44**. The characterization data obtained suggested that a major rearrangement of the molecule had taken place, leading to the formation of **45** (Scheme 9, Schemes S8–S10 and Figure S5 for a more detailed discussion of the data used in this assignment). In this case, it was not possible for us to prepare suitable quality crystals of **45**, emphasizing again how, in the absence of X-ray crystallographic analysis, certainty in structural assignment and especially stereochemistry can prove difficult in such complex systems (compound **45** is a single diastereomer but at present the relative stereochemistry at the centers marked * is unknown).

## 3. Experimental

### 3.1. General Experimental Details

Nuclear magnetic resonance (NMR) spectra were recorded on a Bruker Advance 300 (^1^H, 300; ^13^C, 75 MHz), Bruker Advance II 400 (^1^H, 400; ^13^C, 101 MHz) or Bruker Ascend 500 (^1^H 500; ^13^C 126 MHz). ^13^C NMR spectra were recorded using the PENDANT pulse sequence. Peaks were assigned where possible with the aid of the two-dimensional NMR spectroscopic techniques COSY, HSQC, and HMBC. All NMR spectra were acquired using the deuterated solvent as the lock and the residual solvent as the internal reference. Melting points were recorded in open capillaries using an Electrothermal 9100 melting point apparatus. Values are quoted to the nearest 1 °C and are uncorrected. Infrared spectra were recorded on a Perkin Elmer Paragon 1000 FT spectrometer. Absorption maxima are reported in wavenumbers (cm^−1^). Mass spectra were recorded using either atmospheric pressure chemical ionization (APCI) or electrospray (ES) ionization methods in the positive or negative ionization mode by Mrs Caroline Horsburgh in the University of St Andrews School of Chemistry mass spectrometry service or via the EPSRC Mass Spectrometry Service Centre (Swansea, UK).

### 3.2. X-ray Structure Determination for ***27*** and ***41***

X-ray diffraction data for compounds **27** and **41** were collected at 93 K using a Rigaku MM007 High Brilliance RA generator/confocal optics and Mercury70 CCD system [Mo Kα radiation (λ = 0.71073 Å)]. Intensity data were collected using both ω and φ steps accumulating area detector images spanning at least a hemisphere of reciprocal space. Data for all compounds analyzed were collected and processed (including correction for Lorentz, polarization and absorption) using CrystalClear [46]. Structures were solved by Patterson methods (PATTY [47]) and refined by full-matrix least-squares against F^2^ (SHELXL-2018/3 [48]). Non-hydrogen atoms were refined anisotropically, and hydrogen atoms were refined using a riding model. All calculations were performed using the CrystalStructure [49] interface. Selected crystallographic data are presented in Table S3. Deposition numbers 2109434 and 2109435 contain the supplementary crystallographic data for this paper. These data are provided free of charge by the joint Cambridge Crystallographic Data Center and Fachinformationszentrum Karlsruhe Access Structures service www.ccdc.cam.ac.uk/structures.

### 3.3. Preparation of Selected Compounds

#### 3.3.1. N-Boc-5,7-dichloroindole-3-carbaldehyde **16**

To a suspension of **15** (5.00 g, 23.36 mmol) and Boc_2_O (5.61 g, 25.69 mmol) in DCM (100 mL) was added DMAP (0.342 g, 2.80 mmol). After stirring for 15 min, a saturated solution of NaHCO_3_ (aq.) (100 mL) was added and the reaction mixture extracted with DCM (2 × 50 mL). The organic phase was washed with 0.5 M HCl (aq.) (100 mL) before being dried (MgSO_4_), filtered and the solvent removed under reduced pressure to give **16** as a pale yellow solid that required no further purification (6.93 g, 94%). m.p. 102–103 °C; I.R. (KBr) ν_max_ 2924, 1723, 1601, 1465, 1453, 1253, 735 cm^-1^; ^1^H NMR (400 MHz, CDCl_3_) δ 10.07 (s, 1H, CHO), 8.30 (d, *J* = 2.0 Hz, 1H, C4-H), 8.20 (s, 1H, C2-H), 7.46 (d, *J* = 2.0 Hz, 1H, C6-H), 1.71 (s, 9H, (CH_3_)_3_); ^13^C NMR (100 MHz, CDCl_3_) 184.8 (CHO), 147.5 (CO_2_*^t^*Bu), 140.0 (C2), 131.3 (C7a), 130.9 (C3a), 129.9 (C5), 128.0 (C6), 120.7 (C7), 120.6 (C4), 120.2 (C3), 86.9 (C(CH_3_)_3_), 27.8 (C(CH_3_)_3_); HR MS [ES^+^]: *m/z* calcd. for C_14_H_13_^35^Cl_2_NO_3_Na 336.0170, found 336.0164 [M+Na]^+^.

#### 3.3.2. tert-Butyl 3-((4-bromo-2-nitrophenyl)(hydroxy)methyl)-5,7-dichloro-1*H*-indole-1-carboxylate **19**

To a solution of **17** (5.16 g, 15.70 mmol) in THF (23 mL) at −40 °C was added 2 M solution of phenylmagnesium chloride in THF (8.40 mL, 16.80 mmol), followed 15 min later by a solution of **16** (3.30 g, 10.50 mmol) in THF (23 mL). The mixture was stirred for 1 h at −40 °C before being allowed to warm to room temperature for a further 1 h. A saturated solution of NH_4_Cl (aq.) (50 mL) was then added followed by water (75 mL) and ethyl acetate (75 mL). The mixture was separated and the organic phase washed with a saturated solution of NaCl (aq.) (50 mL), dried (MgSO_4_) and the solvent removed under reduced pressure. The crude product was purified by column chromatography (10–20% EtOAc/hexanes), giving **19** as a yellow solid (5.21 g, 96%). m.p. 56–58 °C; I.R. (KBr) ν_max_ 3386, 1737, 1531, 1346, 1150 cm^-1^; ^1^H NMR (400 MHz, CDCl_3_) δ 8.08 (d, *J* = 2.0 Hz, 1H, C3′-H), 7.70 (dd, *J* = 8.4, 2.0 Hz, 1H, C5′-H), 7.57 (d, *J* = 8.4 Hz, 1H, C6′-H), 7.30 (s, 1H, C2-H), 7.29 (d, *J* = 1.9 Hz, 1H, ArC-H), 7.26 (d, *J* = 1.9 Hz, 1H, ArC-H), 6.50 (d, *J* = 4.4 Hz, 1H, CHOH), 2.84 (d, *J* = 4.4 Hz, 1H, OH), 1.56 (s, 9H, (CH_3_)_3_); ^13^C NMR (100 MHz, CDCl_3_) δ 148.5 (CO_2_*^t^*Bu), 148.3 (C2′), 136.8 (C5′), 135.8 (C1′), 132.2 (ArC), 131.3 (ArC), 130.6 (C6′), 129.0 (ArC), 128.8 (C2), 128.0 (C3′), 126.8 (ArC), 122.4 (C4′), 121.2 (ArC), 119.9 (ArC), 118.1 (ArC), 85.5 (C(CH_3_)_3_), 65.0 (CHOH), 27.8 (C(CH_3_)_3_); HR MS [APCI^+^]: *m/z* calcd. for C_20_H_17_^79^Br^35^Cl_2_N_2_O_5_NH_4_ 532.0036, found 532.0034 [M+NH_4_]^+^.

#### 3.3.3. tert-Butyl 3-(4-bromo-2-nitrobenzoyl)-5,7-dichloro-1*H*-indole-1-carboxylate **20**

To a solution of **19** (3.25 g, 6.30 mmol) and *N*-methylmorpholine-*N*-oxide (1.48 g, 12.60 mmol) in DCM (35 mL) in the presence of 3Å molecular sieves at 0 °C was added tetrapropylammonium perruthenate (0.110 g, 0.310 mmol). The mixture was stirred for 30 min at 0 °C followed by a further 6 h at room temperature before the mixture was filtered and the solvent removed under reduced pressure. The crude product was purified by column chromatography (5–25% EtOAc/hexanes), giving **20** as a white solid (2.31 g, 71%). m.p. 126-127 °C; I.R. (KBr) ν_max_ 2924, 1745, 1654, 1544, 1348 cm^-1^; ^1^H NMR (400 MHz, CDCl_3_) δ 8.42 (d, *J* = 1.9 Hz, 1H, C4-H), 8.39 (d, *J* = 1.8 Hz, 1H, C3′-H), 7.95 (dd, *J* = 8.1, 1.9 Hz, 1H, C5′-H), 7.64 (s, 1H, C2-H), 7.48 (d, *J* = 2 Hz, 1H, C6-H), 7.47 (d, *J* = 8.1 Hz, 1H, C6′-H), 1.66 (s, 9H, C(CH_3_)_3_); ^13^C NMR (100 MHz, CDCl_3_) δ 186.2 (C=O), 147.9 (CO_2_*^t^*Bu), 147.3 (C2′), 137.5 (C2), 137.0 (C5′), 134.5 (C1′), 131.1 (C7a), 131.1 (ArC), 130.9 (ArC), 130.0 (C6′), 128.0 (C6), 128.0 (C3′), 124.5 (C4′), 121.1 (C4), 120.7 (ArC), 118.6 (C3a), 87.5 (C(CH_3_)_3_), 27.7 (C(CH_3_)_3_); HR MS [ES^+^]: *m/z* calcd. for C_20_H_15_^79^Br^35^Cl_2_N_2_O_5_ 534.9439, found 534.9432 [M+H]^+^.This was prepared as in 3.3.1. using benzotriazole (14.69 g, 123.3 mmol), thionyl chloride (3.67 g, 2.24 mL, 30.8 mmol) and (*E*)-3-(2-methylphenyl)prop-2-enoic acid (5.00 g, 30.8 mmol). Drying and evaporation followed by recrystallization of the residue (CH_2_Cl_2_) gave **33** (1.77 g, 22%) as a white powder, m.p. 124–125 °C (Lit. [21] 127–129 °C); δ_H_ 8.47 (1 H, d, *J* 15, COC*H*=CH), 8.42 (1 H, dt, *J* 8, 1, H-4 or 7 of Bt), 8.16 (1 H, dt, *J* 8, 1, H-4 or 7 of Bt), 8.07 (1 H, d, *J* 15, COCH=C*H*), 7.86 (1 H, d, *J* 8), 7.69 (1 H, ddd, *J* 8, 7, 1, H-5 or 6 of Bt), 7.54 (1 H, ddd, *J* 8, 7, 1, H-5 or 6 of Bt), 7.40–7.27 (3 H, m) and 2.56 (3 H, s, Me).

#### 3.3.4. (2-Amino-4-bromophenyl)(5,7-dichloro-1*H*-indol-3-yl)methanone **21**

To a solution of **22** (800 mg, 1.85 mmol) in DCM (9 mL) was added trifluoroacetic acid (3 mL) and the solution was stirred at room temperature for 18 h. The solvent was removed under reduced pressure before a saturated solution of NaHCO_3_ (aq.) (20 mL) was added and the mixture extracted with DCM (3 × 20 mL). The organic extracts were dried (MgSO_4_), filtered and the solvent removed under reduced pressure to give **21** as an orange solid (673 mg, 94%) that required no further purification. m.p. 191–193 °C; I.R. (KBr) ν_max_ 3446, 3346, 1691, 1597, 1428, 1189 cm^−1^; ^1^H NMR (400 MHz, d^6^-DMSO) δ 12.65 (s, 1H, NH), 8.09 (d, *J* = 1.9 Hz, 1H, C4-H), 7.94 (d, *J* = 3.1 Hz, 1H, C2-H), 7.53 (d, *J* = 8.4 Hz, 1H, C6′-H), 7.46 (d, *J* = 1.8 Hz, 1H, C6-H), 7.03 (d, *J* = 1.9 Hz, 1H, C3′-H), 6.73 (dd, *J* = 8.4, 1.9 Hz, 1H, C5′-H), 6.64 (br. s, 2H, NH_2_); ^13^C NMR (100 MHz, d^6^-DMSO) δ 190.3 (C=O), 151.0 (C2′), 136.0 (C2), 133.5 (C6′), 132.6 (C7a), 129.2 (C3a), 126.7 (C5), 126.6 (C3), 126.3 (C4′), 122.2 (C6), 119.6 (C4), 119.1 (C1′), 118.5 (C3′), 117.6 (C5′) 116.8 (C7); HR MS (ES^-^) *m/z* calcd. for C_15_H_8_^79^Br^35^Cl_2_N_2_O 380.9197, found 380.9192 [M-H]^−^.

#### 3.3.5. tert-Butyl 3-(2-amino-4-bromobenzoyl)-5,7-dichloro-1*H*-indole-1-carboxylate **22**

A suspension of **20** (1.00 g, 1.945 mmol) and iron powder (0.543 g, 9.725 mmol) in acetic acid (10 mL) and ethanol (10 mL) was stirred for 18 h at room temperature. The mixture was filtered through a plug of Celite. The celite was washed with DCM (50 mL) and combined with the existing filtrate. Water (50 mL) was added. The mixture was partitioned and the aqueous layer further extracted with DCM (2 × 25 mL). The combined organic layers were concentrated under reduced pressure before being re-dissolved in DCM (50 mL), washed with a saturated solution of NaHCO_3_ (aq.) (100 mL), dried (MgSO_4_), filtered and the solvent removed under reduced pressure. The crude product was purified by column chromatography (10–25% EtOAc/hexanes) to give **22** as a yellow solid (0.876 g, 93%). m.p. 143–144 °C; I.R. (KBr) ν_max_ 3442, 3336, 1735, 1645, 1605 cm^−1^; ^1^H NMR (400 MHz, d^6^-DMSO) δ 8.01 (d, *J* = 2.0 Hz, 1H, C4-H), 7.80 (s, 1H, C2-H), 7.44 (d, *J* = 8.5 Hz, 1H, C6′-H), 7.35 (d, *J* = 1.9 Hz, 1H, C6-H), 6.87 (d, *J* = 1.8 Hz, 1H, C3′-H), 6.75 (dd, *J* = 8.4, 1.9 Hz, 1H, C5′-H), 5.87 (br. s, 2H, NH_2_), 1.58 (s, 9H, C(CH_3_)_3_); ^13^C NMR (100 MHz, d^6^-DMSO) δ 190.8 (C=O), 150.9 (C2′), 147.0 (CO_2_*^t^*Bu), 135.7 (C2), 133.7 (C6′), 132.4 (C7a), 130.3 (C), 130.0 (C), 128.9 (C4′), 127.4 (C6), 120.8 (C), 120.6 (C4), 119.5 (C3′), 119.3 (C5′), 119.3 (C), 118.6 (C1′), 86.6 (C(CH_3_)_3_), 27.8 (C(CH_3_)_3_); HR MS [ES^+^]: *m/z* calcd. for C_20_H_17_^79^Br^35^Cl_2_N_2_O_3_Na 504.9697, found 504.9693 [M+Na]^+^.

#### 3.3.6. (2-Benzylamino)-4-bromophenyl)(5,7-dichloro-1*H*-indol-3-yl)methanone **9**

To a solution of **22** (1.00 g, 2.07 mmol) in TFA (8 mL) was added sodium triacetoxyborohydride (0.875 g, 4.13 mmol). After 5 min, a solution of benzaldehyde (0.219 g, 2.07 mmol) in DCM (8 mL) was added dropwise and the solution was stirred for 1 h at room temperature. A second aliquot of sodium triacetoxyborohydride (0.875 g, 4.13 mmol) in TFA (4mL) was added and the mixture was stirred for a further 1 h at room temperature. The mixture was then poured into an ice cold saturated solution of NaHCO_3_ (aq.) (20 mL) and extracted with DCM (3 × 10 mL). The combined organic extracts were dried (MgSO_4_), filtered and the solvent removed under reduced pressure before the crude product was purified by column chromatography (5-30% EtOAc/hexanes), giving **22** as an orange-yellow solid (0.558 g, 68%). m.p. 81–82 °C; I.R. (KBr) ν_max_ 1691 (C=O), 1602, 1419, 1185 cm^−1^; ^1^H NMR (400 MHz, CDCl_3_) δ 8.81 (br. s, 1H, NH), 8.28 (br. t, *J* = 5.3 Hz, 1H, NHBn), 8.05 (d, *J* = 1.5 Hz, 1H, C4-H), 7.54 (d, *J* = 2.9 Hz, 1H, C2-H), 7.48 (d, *J* = 8.4 Hz, 1H, C6′-H), 7.30–7.19 (m, 6H, C6-H and 5 x ArC-H), 6.83 (d, *J* = 1.8 Hz, 1H, C3′-H), 6.68 (dd, *J* = 8.4, 1.9 Hz, 1H, C5′-H), 4.34 (d, *J* = 5.5 Hz, 2H, CH_2_); ^13^C NMR (100 MHz, CDCl_3_) δ 191.6 (C=O), 151.0 (C2′), 137.9 (ArC), 134.1 (C6′), 132.6 (C2), 132.1 (C7a), 129.2 (C4′), 128.8 (ArCH), 128.4 (C3a), 128.3 (C3), 127.5 (ArCH), 127.3 (ArCH), 123.5 (C6), 120.5 (C4), 118.9 (ArC), 118.5 (C1′), 117.8 (C5′), 117.3 (C), 114.7 (C3′), 47.2 (CH_2_); HR MS [ES^-^]: *m/z* calcd. for C_22_H_14_^79^Br^35^Cl_2_N_2_O 470.9667, found 470.9673 [M-H]^−^. **21** was also obtained as an orange solid (0.151 g, 19%).

#### 3.3.7. 5-Benzyl-3-bromo-7,9-dichloro-5*H*-indolo[2,3-*b*]quinolin-11(6*H*)-one **3**

To a solution of **9** (250 mg, 0.527 mmol) in MeCN (6 mL) was added *N*-chlorosuccinimide (169 mg, 1.265 mmol) and Et_3_N (176 μL, 1.265 mmol). The mixture was stirred for 24 h at room temperature before the precipitate was collected by filtration, giving **3** as a cream-colored solid (137 mg, 55%). m.p. >320 °C (dec.); I.R. (KBr) ν_max_ 1706, 1603, 1536, 1181 cm^−1^; ^1^H NMR (400 MHz, d6-DMSO) δ 12.45 (br. s, 1H, NH), 8.27 (d, *J* = 8.5 Hz, 1H, C1-H), 8.14 (d, *J* = 1.9 Hz, 1H, C10-H), 7.78 (d, *J* = 1.6 Hz, 1H, C4-H), 7.54 (dd, *J* = 8.5, 1.6 Hz, 1H, C2-H), 7.46 (d, *J* = 1.9 Hz, 1H, C8-H), 7.36–7.26 (m, 3H, 3 x ArC-H), 7.18–7.16 (m, 2H, 2 × ArC-H), 5.98 (s, 2H, CH_2_); ^13^C NMR (100 MHz, d6-DMSO) δ 171.4 (C=O), 148.0 (C5a), 139.6 (C4a), 135.6 (ArC), 130.6 (C6a), 129.0 (ArCH), 127.9 (C1), 127.5 (ArCH), 126.5 (ArC), 126.3 (ArC), 126.0 (ArCH), 125.6 (C2), 125.5 (C3), 123.8 (C11a), 122.4 (C8), 118.8 (C4), 118.0 (C10), 116.2 (ArC), 49.0 (CH_2_); HR MS [ES^+^]: *m/z* calcd. for C_22_H_14_^35^Cl_2_^81^BrN_2_O 470.9661, found 470.9659 [M+H]^+^.

#### 3.3.8. 5-Benzyl-3-bromo-7,9,11-trichloro-5*H*-indolo[2,3-*b*]quinoline **25**

A solution of **3** (1.50 g, 3.18 mmol) in POCl_3_ (15 mL) was heated to reflux for 1 h before removing the solvent under reduced pressure. DCM (100 mL) and a saturated solution of NaHCO_3_ (aq.) (250 mL) were added and the mixture partitioned before the organic layer was dried (MgSO_4_), filtered and concentrated to give **25** in sufficient purity without further purification (1.54 g, 99%). m.p. 260–263 °C; I.R. (KBr) ν_max_ 1705, 1602, 1484, 1175, 847 cm^-1^; ^1^H NMR (400 MHz, CDCl_3_) δ 8.31 (d, *J* = 2.0 Hz, 1H, C10-H), 8.28 (d, *J* = 8.8 Hz, 1H, C1-H), 7.87 (d, *J* = 1.7 Hz, 1H, C4-H), 7.59 (d, *J* = 1.7 Hz, 1H, C2-H), 7.58 (d, *J* = 2.0 Hz, 1H, C8-H), 7.36–7.27 (m, 5H, 5 × Ar-H), 6.20 (s, 2H, CH_2_); ^13^C NMR (101 MHz, CDCl_3_) δ 155.4 (C5a), 150.3 (C6a), 137.2 (ArC), 134.8 (ArC), 129.3 (C8), 129.1 (ArCH), 128.1 (ArCH), 127.7 (C1), 127.0 (ArCH), 126.9 (ArC), 126.3 (C2), 125.9 (ArC), 125.6 (ArC), 124.4 (ArC), 123.2 (ArC), 122.0 (C10), 118.5 (C4), 118.3 (ArC), 50.0 (CH_2_); HR MS (ES^+^) *m/z* calcd. for C_22_H_12_N_2_^35^Cl_3_^81^BrNa 512.9127, found 512.9130 [M+Na]^+^.

#### 3.3.9. 11-(Allyloxy)-5-benzyl-3-bromo-7,9-dichloro-5*H*-indolo[2,3-*b*]quinoline **26**

To a suspension of sodium (77 mg, 3.36 mmol) in THF (2 mL) at room temperature was added allyl alcohol (0.76 mL, 11.21 mmol) dropwise to maintain a steady reaction. Once all of the sodium had dissolved, the alkoxide solution was added via cannula to a stirred solution of **25** (550 mg, 1.12 mmol) in THF (11 mL) and the mixture was stirred at room temperature for 2 h. A saturated solution of NH_4_Cl (aq.) (10 mL) was added. The solvent was removed under reduced pressure and the crude residue was extracted with DCM (3 × 30 mL). The combined organic extracts were dried (MgSO_4_) filtered and concentrated under reduced pressure, giving **26** in sufficient purity without any further purification as an orange solid (525 mg, 91%). m.p. 225–227 °C; I.R. (KBr) ν_max_ 2925, 1641, 1561, 1487, 1177, 858 cm^−1^; ^1^H NMR (300 MHz, CDCl_3_) δ 8.18 (d, *J* = 8.7 Hz, 1H, Ar-H), 7.99 (d, *J* = 2.0 Hz, 1H, Ar-H), 7.86 (d, *J* = 1.6 Hz, 1H, Ar-H), 7.56 (d, *J* = 1.9 Hz, 1H, Ar-H), 7.53 (dd, *J* = 8.7, 1.7 Hz, 1H, Ar-H), 7.37–7.27 (m, 5H, 5 × Ar-H), 6.32–6.20 (m, 1H, CH_2_=CH), 6.19 (s, 2H, CH_2_N), 5.56 (dq, *J* = 17.1, 1.4 Hz, 1H, CH_2_=CH), 5.43 (dq, *J* = 10.4, 1.1 Hz, 1H, CH_2_=CH), 4.99 (dt, *J* = 5.7, 1.3 Hz, 2H, CH_2_); ^13^C NMR (75 MHz, CDCl_3_) δ 158.7 (C5a), 158.4 (C11), 149.4 (ArC), 148.9 (ArC), 138.6 (C4a), 135.2 (ArC), 132.0 (CH=CH_2_), 129.1 (ArCH), 128.0 (ArCH), 127.9 (ArCH), 127.0 (ArCH), 126.5 (C), 126.1 (ArCH), 125.6 (ArCH), 125.2 (ArC), 124.7 (ArC), 121.3 (ArCH), 119.8 (CH=CH_2_), 118.5 (ArCH), 117.0 (ArC), 116.0 (ArC), 76.1 (CH_2_-C=C), 49.7 (CH_2_N); HR MS (ES^+^) *m/z* calcd. for C_25_H_18_^79^Br^35^Cl_2_N_2_O 510.9980, found 510.9979 [M+H]^+^.

#### 3.3.10. 10b-Allyl-5-benzyl-3-bromo-7,9-dichloro-5*H*-indolo[2,3-*b*]quinolin-11(10b*H*)-one **27**

A solution of **26** (500 mg, 0.98 mmol) in toluene (10 mL) was heated to reflux for 1.5 h before removing the solvent under reduced pressure. The crude product was purified by column chromatography (10–25 % EtOAc/hexanes) to give **27** as an orange-yellow solid (477 mg, 95%). Recrystallization by slow evaporation of a solution of **27** in EtOAc/Hexane provided crystals suitable for X-ray analysis. m.p. 240 °C (dec.); I.R. ν_max_ 1695, 1543, 1196, 854 cm^-1^; ^1^H NMR (400 MHz, CDCl_3_) δ 7.80 (d, *J* = 8.8 Hz, 1H, C1-H), 7.59 (d, *J* = 2.0 Hz, 1H, C10-H), 7.43–7.37 (m, 5H, 5 × ArC-H), 7.33 (m, 1H, ArC-H), 7.29–7.26 (m, 2H, 2 × ArC-H), 5.98 (d, *J* = 16.4 Hz, 1H, 1 × CH_2_N), 5.38 (ddt, *J* = 16.9, 10.1, 7.3 Hz, 1H, CH=CH_2_), 5.14–5.03 (m, 2H, 1 × CH_2_N, 1 × CH=CH_2_), 4.90 (dd, *J* = 16.8, 1.3 Hz, 1H, CH=CH_2_), 2.87 (dd, *J* = 13.4, 6.9 Hz, 1H, CH_2_), 2.53 (dd, *J* = 13.4, 7.7 Hz, 1H, CH_2_); ^13^C NMR (101 MHz, CDCl_3_) δ 190.4 (C11), 172.2 (C5a), 149.0 (C6a), 145.1 (C4a), 135.7 (C10a), 135.4 (C), 131.1 (C3), 129.7 (C1), 129.2 (ArCH), 129.1 (ArCH), 129.1 (ArC), 128.9 (C10b^II^), 128.0 (ArCH), 126.8 (ArCH), 126.4 (ArCH), 123.9 (ArC), 123.5 (C10), 121.6 (C10b^III^), 118.9 (ArCH), 117.6 (ArC), 67.0 (C10b), 49.9 (C10b^I^), 44.8 (CH_2_N); **HR MS** (ES^+^) *m/z* calcd. for C_25_H_17_^79^Br^35^Cl^37^ClN_2_ONa 534.9769, found 534.9767 [M+Na]^+^.

#### 3.3.11. (*E*)-5-Benzyl-3-bromo-10b-(but-2-en-1-yl)-7,9-dichloro-5*H*-indolo[2,3-*b*]quinolin-11(10b*H*)-one **29**

To a suspension of sodium (205 mg, 8.93 mmol) in THF (5 mL) at room temperature was added (±)-3-buten-2-ol (2.58 mL, 29.76 mmol) dropwise to maintain a steady reaction. Once all of the sodium had dissolved, the alkoxide solution was added via a cannula to a stirred solution of **25** (1.46 g, 2.98 mmol) in THF (30 mL) and the mixture was stirred at room temperature for 2 h. A saturated solution of NH_4_Cl (aq.) (30 mL) was added before the organic solvent was removed under reduced pressure and the crude residue extracted with DCM (3 × 100 mL). The combined organic extracts were dried (MgSO_4_), filtered and concentrated under reduced pressure to give a crude product that contained **28** and **29**. This residue was redissolved in THF (30 mL) and the solution was heated at reflux for 1 h before removing the solvent under reduced pressure to give the crude product. Purification by column chromatography (10–25% EtOAc/hexanes) gave **29** as a yellow solid (1.17 g, 75%). m.p. 140–142 °C; ν_max_ 1698, 1590, 1545, 1196, 855 cm^−1^; ^1^H NMR (500 MHz, CDCl_3_) δ 7.71 (d, *J* = 8.6 Hz, 1H, C1-H), 7.48 (d, *J* = 2.0 Hz, 1H, C10-H), 7.36–7.14 (m, 8H, 8 × ArC-H), 5.91 (d, *J* = 16.3 Hz, 1H, CH_2_N), 5.21 (dq, *J* = 12.6, 5.8 Hz, 1H, C10b^II^-H), 5.01–4.88 (m, 2H, CH_2_N + C10b^III^-H), 2.74 (dd, *J* = 13.2, 6.4 Hz, 1H, C10b^I^-H_2_), 2.35 (dd, *J* = 13.2, 8.1 Hz, 1H, C10b^I^-H_2_), 1.49 (d, *J* = 6.2 Hz, 3H, C10b^IV^-H_3_); ^13^C NMR (126 MHz, CDCl_3_) δ 190.5 (C11), 172.4 (C5a), 148.9 (C6a), 145.1 (C4a), 136.0 (C10a), 135.5 (C), 132.9 (C10b^II^), 131.0 (C3), 129.6 (C1), 129.1 (ArCH), 129.0 (ArCH), 128.9 (ArC), 128.0 (ArCH), 126.8 (ArCH), 126.3 (ArCH), 123.8 (ArC), 123.6 (C10), 121.4 (C10b^III^), 118.8 (C8), 117.8 (C11a), 67.4 (C10b), 49.9 (CH_2_N), 44.3 (C10b^I^), 17.9 (C10b^IV^); HR MS (ES^-^) *m/z* calcd. for C_26_H_18_N_2_O^35^Cl_2_^81^Br 524.9959, found 524.9971 [M − H]^−^.

#### 3.3.12. 5-Benzy-3-bromo-10b-((*E*)-but-2-en-1-yl)-7,9-dichloro-5,10b-dihydrospiro[indolo[2,3-b]quinoline-11,2′-oxirane] **31**

To a solution of **29** (200 mg, 0.380 mmol) and chloroiodomethane (42 μL, 0.570 mmol) in THF (4 mL) at −78 °C was added methyl lithium—lithium bromide complex (1.5 M in THF, 0.38 mL, 0.570 mmol) dropwise over 5 min. The mixture was stirred at −78 °C for a further 30 min before removing the cold bath and stirring for a further 18 h at room temperature. A solution of saturated NH_4_Cl (aq.) (10 mL) was added and the mixture extracted with DCM (3 × 10 mL) before the combined organic extracts were dried (MgSO_4_), filtered and concentrated. The crude product was purified by column chromatography (10–20 % EtOAc/hexanes) to give **31** as an orange solid (142 mg, 74%). m.p. 90–93 °C; I.R. (KBr) ν_max_ 2921, 1709, 1595, 1550, 1486, 729 cm^−1^; ^1^H NMR (400 MHz, CDCl_3_) δ 7.41–7.35 (m, 4H, 4 × ArC-H), 7.33–7.28 (m, 2H, 2 × ArC-H), 7.19 (dd, *J* = 8.1, 1.7 Hz, 1H, C2-H), 7.12 (d, *J* = 1.7 Hz, 1H, C4-H), 7.11–7.05 (m, 2H, 2 × ArC-H), 5.89 (d, *J* = 16.2 Hz, 1H, CH_2_N), 5.37–5.22 (m, 1H, C10b^III^-H), 4.97–4.86 (m, 2H, CH_2_N + C10b^II^-H), 3.03 (d, *J* = 5.3 Hz, 1H, CH_2_O), 2.76 (ddt, *J* = 13.9, 6.1, 1.3 Hz, 1H, C10b^I^-H_2_), 2.59 (ddt, *J* = 13.9, 6.1, 1.3 Hz, 1H, C10b^I^-H_2_), 2.52 (d, *J* = 5.3 Hz, 1H, CH_2_O), 1.50 (d, *J* = 6.0 Hz, 3H, CH_3_); ^13^C NMR (101 MHz, CDCl_3_) δ 172.6 (C5a), 151.0 (C6a), 142.0 (C4a), 137.2 (C10a), 136.1 (ArC), 130.8 (C10b^III^), 129.1 (ArCH), 129.0 (ArCH), 128.4 (ArC), 127.7 (ArCH), 126.9 (ArCH), 126.5 (ArCH), 125.2 (ArCH), 123.3 (ArC), 123.1 (ArC), 122.9 (C10b^II^), 122.1 (C11a), 121.5 (ArCH), 118.4 (ArCH), 59.5 (C11), 56.4 (C10b), 53.2 (CH_2_O), 50.0 (CH_2_N), 37.1 (C10b^I^), 17.8 (C10b^IV^); HR MS [APCI^+^]: *m/z* calcd. for C_27_H_22_^79^Br^35^Cl_2_N_2_O 539.0287, found 539.0275 [M+H]^+^.

#### 3.3.13. (10b*R*,11*R*)-5-Benzyl-3-bromo-10b-((*E*)-but-2-en-1-yl)-7,9-dichloro-10b,11-dihydro-5*H*-indolo[2,3-*b*]quinolin-11-yl)methanol **32**

To a solution of **31** (90 mg, 0.167 mmol) in THF (3 mL) at -78 °C was added sodium cyanoborohydride (26 mg, 0.416 mmol) and boron trifluoride diethyl etherate (82μL, 0.666 mmol). The mixture was slowly allowed to warm to room temperature over a period of 6 h before a saturated solution of NaHCO_3_ (aq.) (6 mL) was added. The mixture was extracted with DCM (3 × 10 mL) before the combined organic extracts were dried (MgSO_4_), filtered and concentrated to give the crude product. The crude reaction mixture was purified by column chromatography (20–30 % EtOAc/hexanes) to give **32** as a white solid (45 mg, 50%). m.p. 85–86 °C, I.R. (KBr) ν_max_ 3419 (OH), 2931, 1545, 1486, 1422, 1206 cm^-1^; ^1^H NMR (300 MHz, CDCl_3_) δ 7.54 (dd, *J* = 8.3, 1.0 Hz, 1H, C2-H), 7.38–7.24 (m, 6H, 6 × ArC-H), 7.23–7.16 (m, 2H, 2 × ArC-H), 7.10 (d, *J* = 1.9 Hz, 1H, ArC-H), 5.75 (d, *J* = 16.2 Hz, 1H, CH_2_N), 5.19 (dq, *J* = 13.0, 6.5 Hz, 1H, CH=CHCH_3_), 4.80 (d, *J* = 16.2 Hz, 1H, CH_2_N), 4.72–4.58 (m, 1H, CH=CHCH_2_), 4.53 (dd, *J* = 11.2, 2.4 Hz, 1H, CH_2_OH), 4.39–4.26 (m, 1H, CH_2_OH), 3.00 (d, *J* = 6.2 Hz, 1H, C11-H), 2.42–2.26 (m, 2H, CH_2_), 2.14 (br., s, 1H, OH), 1.40 (d, *J* = 6.5 Hz, 3H, CH_3_); ^13^C NMR (75 MHz, CDCl_3_) δ 174.4 (C5a), 151.9 (C6a), 142.3 (C4a), 140.1 (C10a), 136.7 (ArC), 130.6 (C10b^III^), 129.2 (2 × overlapping ArCH), 129.1 (C1), 128.0 (ArCH), 127.5 (ArCH), 126.9 (C2), 125.0 (ArC), 124.5 (C11a), 123.8 (C10b^II^), 123.1 (ArC), 122.9 (ArCH), 122.1 (C3), 119.1 (C4), 61.7 (CH_2_OH), 56.2 (C10b), 50.8 (CH_2_N), 44.0 (C11), 33.9 (C10b^I^), 18.2 (C10b^IV^); HR MS [ES^+^]: *m/z* calcd. for C_27_H_23_^79^Br^35^Cl^37^ClN_2_ONa 565.0239, found 565.0220 [M+Na]^+^.

#### 3.3.14. (10b*R*,11*R*)-11-Allyl-5-benzyl-3-bromo-7,9-dichloro-10b-(2-hydroxyethyl)-10b,11-dihydro-5*H*-indolo[2,3-b]quinolin-11-ol **40**

For details of the synthesis and analysis of the diastereomeric mixtures **37a**/**37b**, **38a**/**38b** and **39a**/**39b** see SI. To a solution of **39a**/**39b** (230 mg, 0.413 mmol) in MeOH (10 mL) was added NaBH_4_ (31 mg, 0.826 mmol) and the mixture was stirred at room temperature for 1 h. A saturated solution of NH_4_Cl (aq.) (10 mL) was added and the organic solvent was removed under reduced pressure before the mixture was extracted with DCM (3 × 10 mL). The combined organic extracts were dried (MgSO_4_), filtered and concentrated in vacuo. The crude product was purified by column chromatography (15-25% EtOAc/hexanes) to give **40** as a white solid (220 mg, 95%). m.p. = 151–153 °C; I.R. (KBr) ν_max_ 3163, 2931, 1545, 1481, 1418, 1206, 847, 729 cm^−1^; ^1^H NMR (500 MHz, CDCl_3_) δ 7.40–7.31 (m, 6H, C8-H, C10-H, 4 × ArC-H), 7.30–7.25 (m, 2H, C1-H, ArC-H), 7.19 (dd, *J* = 8.1, 1.7 Hz, 1H, C2-H), 7.13 (d, *J* = 1.7 Hz, 1H, C4-H), 5.56 (d, *J* = 16.1 Hz, 1H, CH_2_N), 5.38 (ddt, *J* = 17.3, 10.0, 7.3 Hz, 1H, C11^II^-H), 5.22 (d, *J* = 16.0 Hz, 1H, CH_2_N), 4.98–4.92 (m, 1H, C11^III^-H_2_), 4.73 (dt, *J* = 17.0, 1.6 Hz, 1H, C11^III^-H_2_), 3.60 (ddd, *J* = 12.0, 8.7, 3.6 Hz, 1H, C10b^II^-H_2_), 3.52–3.45 (m, 1H, C10b^II^-H_2_), 2.41–2.32 (m, 1H, C10b^I^-H_2_), 2.12 (d, *J* = 7.0 Hz, 2H, C11^I^-H_2_), 1.78–1.69 (m, 1H, C10b^I^-H_2_); ^13^C NMR (126 MHz, CDCl_3_) δ 175.4 (C5a), 150.9 (C6a), 138.9 (C4a), 137.9 (C10a), 135.8 (ArC), 131.6 (C11^II^), 129.2 (C8), 128.9 (2 x overlapping carbons ArCH, C11a), 128.4 (ArCCl), 128.2 (C1 or ArCH), 127.9 (C1 or ArCH), 127.3 (ArCH), 126.4 (C2), 122.9 (ArCCl), 122.7 (C10), 122.3 (C3), 120.0 (C11^III^), 118.6 (C4), 75.3 (C11), 61.3 (C10b), 58.6 (C10b^II^), 49.4 (CH_2_N), 40.6 (C11^I^), 35.0 (C10b^I^). HR MS [ES^+^]: *m/z* calcd. for C_27_H_24_^79^Br^35^Cl_2_N_2_O_2_ 557.0393, found 557.0392 [M+H]^+^.

#### 3.3.15. (3a*R*,13b*R*)-13b-Allyl-9-benzyl-11-bromo-5,7-dichloro-2,3,9,13b-tetrahydrofuro[3,2-*c*]indolo [2,3-*b*]quinoline **41**

To a solution of **40** (50 mg, 0.090 mmol) in DCM (2 mL) was added *p*-toluenesulfonyl chloride (26 mg, 0.134 mmol) and triethylamine (124 μL, 0.896 mmol) and the mixture was stirred at room temperature for 1 h. The mixture was then heated to reflux for a further 5 h before cooling to room temperature and addition of a saturated solution of NH_4_Cl (aq.) (5 mL). The mixture was extracted with DCM (3 × 5 mL) and the combined organic extracts were dried (MgSO_4_), filtered and concentrated in vacuo. The crude product was purified by column chromatography (5–15% EtOAc/hexanes) to give **41** as a white solid (40 mg, 83%). Recrystallization of **41** by slow evaporation from ethyl acetate gave crystals of suitable quality for X-ray crystallographic analysis. m.p. = 177–179 °C; I.R. (KBr) ν_max_ 2926, 1548, 1484, 1425, 1204, 1071, 1044, 850 cm^-1^; ^1^H NMR (500 MHz, CDCl_3_) δ 7.29–7.15 (m, 7H, C6-H, C13-H, 5 × ArC-H), 7.14 (d, *J* = 2.0 Hz, 1H, C4-H), 7.10 (dd, *J* = 8.2, 1.8 Hz, 1H, C12-H), 7.00 (d, *J* = 1.8 Hz, 1H, C10-H), 5.62 (d, *J* = 16.1 Hz, 1H, CH_2_N), 5.06–4.95 (m, 2H, CH_2_N, C15-H), 4.67–4.60 (m, 1H, C16-H_2_), 4.39 (dq, *J* = 16.9, 1.3 Hz, 1H, C16-H_2_), 4.12 (q, *J* = 8.3 Hz, 1H, C2-H_2_), 3.74 (ddd, *J* = 10.1, 8.6, 3.4 Hz, 1H, C2-H_2_), 2.49 (ddd, *J* = 12.6, 10.1, 8.2 Hz, 1H, C3-H_2_), 2.38–2.35 (m, 2H, 14-H_2_), 2.03 (ddd, *J* = 12.2, 8.2, 3.4 Hz, 1H, C3-H_2_); ^13^C NMR (126 MHz, CDCl_3_) δ 171.6 (C8a), 149.2 (C7a), 139.2 (C3b), 139.0 (C9a), 134.9 (ArC), 128.9 (C15), 127.9 (C6), 127.9 (ArCH), 127.7 (C13 or ArCH), 127.2 (ArCCl), 126.7 (C13 or ArCH), 126.1 (ArCH), 125.5 (C12), 124.8 (C13a), 122.3 (ArCCl), 121.8 (C11), 120.1 (C4), 119.2 (C16), 117.2 (C10), 85.0 (C13b), 63.3 (C2), 59.5 (C3a), 48.3 (CH_2_N), 40.5 (C14), 36.8 (C3); HR MS [ES^+^]: *m/z* calcd. for C_27_H_22_^79^Br^35^Cl_2_N_2_O 539.0287, found 539.0284 [M+H]^+^.

#### 3.3.16. (*R*)-2-(11,11-Diallyl-5-benzyl-3-bromo-7,9-dichloro-10b,11-dihydro-5*H*-indolo[2,3-b]quinolin-10b-yl)ethanol **35**

To a solution of **41** (40 mg, 0.074 mmol) in DCM (1.5 mL) at −78 °C was added allyltrimethylsilane (42 mg, 0.370 mmol) and TiCl_4_ (70 mg, 0.370 mmol) and the mixture was stirred at −78 °C for 4.5 h. Methanol (0.5 mL) was added and the mixture was stirred for an additional 10 min before the cold bath was removed and a saturated solution of NH_4_Cl (aq.) (5 mL) was added. The mixture was extracted with DCM (3 × 5 mL) and the combined organic extracts were dried (MgSO_4_), filtered and concentrated in vacuo. The crude product was purified by column chromatography (15–30% EtOAc/hexanes) to give **35** as a pale yellow solid (34 mg, 79%). m.p. = 73–75 °C; I.R. (KBr) ν_max_ 3389, 3074, 2921, 1607, 1548, 1486, 1422, 1206, 909, 847, 798, 729, 702 cm^-1^; ^1^H NMR (400 MHz, CDCl_3_) δ 7.47–7.39 (m, 2H, 2 × ArC-H), 7.36–7.27 (m, 4H, C8-H, 3 x ArC-H), 7.23 (d, *J* = 1.9 Hz, 1H, C4-H), 7.19 (d, *J* = 1.9 Hz, 1H, C10-H), 7.15 (dd, *J* = 8.3, 1.9 Hz, 1H, C2-H), 7.03 (d, *J* = 8.3 Hz, 1H, C1-H), 6.09 (ddt, *J* = 17.1, 9.6, 4.9 Hz, 1H, C11^IIa^-H), 5.44 (d, *J* = 15.9 Hz, 1H, CH_2_N), 5.38 (d, *J* = 17.1 Hz, 1H, C11^IIIa^-H_2_), 5.28 (d, *J* = 15.9 Hz, 1H, CH_2_N), 5.17 (d, *J* = 10.2 Hz, 1H, C11^IIIa^-H_2_), 5.14–5.03 (m, 1H, C11^IIb^-H), 4.82 (d, *J* = 10.0 Hz, 1H, C11^IIIb^-H_2_), 4.62 (d, *J* = 16.2 Hz, 1H, C11^IIIb^-H_2_), 3.17–3.05 (m, 2H, C10b^II^-H_2_, C11^Ia^-H2), 2.95 (ddd, *J* = 10.6, 8.5, 5.3 Hz, 1H, C10b^II^-H2), 2.75 (dd, *J* = 16.9, 9.2 Hz, 1H, C11^Ia^-H2), 2.34 (ddd, *J* = 13.4, 8.2, 5.3 Hz, 1H, C10b^I^-H_2_), 2.14–2.03 (m, 2H, C10b^I^-H_2_, C11^Ib^-H_2_), 1.80–1.72 (m, 1H, C11^Ib^-H_2_); ^13^C NMR (101 MHz, CDCl_3_) δ 173.9 (C5a), 151.6 (C6a), 140.6 (C4a), 138.9 (C10a), 135.9 (ArCH), 135.8 (C11^IIa^), 132.6 (C11^IIb^), 129.5 (C1), 129.1 (C8), 128.8 (ArCH), 128.1 (ArCCl), 127.7 (ArCH), 126.5 (C11a), 125.9 (C2), 122.8 (ArCCl), 122.2 (C10), 121.6 (C3), 119.5 (C4), 119.2 (C11^IIIb^), 117.1 (C11^IIIa^), 60.5 (C10b), 58.7 (C10b^II^), 49.7 (CH_2_N), 45.9 (C11), 39.9 (C11^Ib^), 37.3 (C11^Ia^), 35.6 (C10b^I^); HR MS [ES^+^]: *m/z* calcd. for C_30_H_28_^79^Br^35^Cl_2_N_2_O 581.0757, found 581.0756 [M+H]^+^.

#### 3.3.17. 3a-(4-Allyl-1-benzyl-7-bromo-2-(iodomethyl)-1,2,3,4-tetrahydroquinolin-4-yl)-5,7-dichloro-3,3a-dihydro-2*H*-furo[2,3-*b*]indole **45**

To a solution of **35** (18 mg, 0.031 mmol) in base-washed CDCl_3_ (1 mL) was added NIS (22 mg, 0.098 mmol) and the mixture was stirred at room temperature for 3 h. A saturated solution of Na_2_S_2_O_3_ (aq.) (2 mL) was added and the mixture was stirred for a further 10 min before being extracted with DCM (3 × 2 mL). The combined organic extracts were dried (MgSO_4_), filtered and concentrated in vacuo. The crude product was purified by column chromatography (Et_3_N washed silica, 5–15% EtOAc/hexanes), giving **45** as a white solid (11 mg, 50%). I.R. (KBr) ν_max_ 3320, 3074, 2921, 1607, 1548, 1486, 1420, 1255, 1206, 911, 847, 798, 729 cm^−1^; ^1^H NMR (500 MHz, CDCl_3_) δ 7.40 (d, *J* = 2.0 Hz, 1H, C6-H), 7.38–7.27 (m, 6H, C4-H, 5 × ArC-H), 7.19 (d, *J* = 8.3 Hz, 1H, C5′-H), 6.93 (dd, *J* = 8.3, 2.0 Hz, 1H, C6′-H), 6.82 (d, *J* = 1.9 Hz, 1H, C8′-H), 5.70 (dddd, *J* = 17.8, 10.1, 7.9, 5.2 Hz, 1H, C4′^II^-H), 5.28–5.17 (m, 2H, C4′^III^-H_2_), 4.64 (d, *J* = 17.9 Hz, 1H, CH_2_N), 4.46 (d, *J* = 17.8 Hz, 1H, CH_2_N), 4.31 (t, *J* = 9.1 Hz, 1H, C2-H_2_), 3.51–3.42 (m, 1H, C2′-H), 3.26 (dd, *J* = 14.4, 5.2 Hz, 1H, C4′^I^-H_2_), 3.07 (ddd, *J* = 10.0, 8.5, 6.3 Hz, 1H, C2-H_2_), 3.01 (dd, *J* = 9.7, 2.7 Hz, 1H, CH_2_I), 2.54 (q, *J* = 7.2 Hz, 3H, 1 × C3-H_2_, 1 x C4′^I^-H_2_, 1 x CH_2_I), 2.09 (dt, *J* = 13.5, 10.0 Hz, 1H, C3-H_2_), 1.58 (dd, *J* = 8.9, 6.7 Hz, 2H, C3′-H_2_); ^13^C NMR (126 MHz, CDCl_3_) δ 193.6 (C8a), 153.8 (C7a), 149.4 (C8a’), 140.3 (C3b), 139.0 (ArC), 132.2 (C4′^II^), 129.5 (C6), 129.1 (C5′), 128.7 (ArCH), 128.4 (ArCCl), 127.2 (ArCH), 126.5 (ArCH), 124.3 (ArCCl), 123.6 (C4), 123.5 (C7′), 120.9 (C4a’), 120.5 (C4′^III^), 120.5 (C6′), 118.3 (C8′), 79.9 (C2), 64.9 (C3a), 57.7 (CH_2_N), 56.8 (C2′), 45.0 (C4′), 38.5 (C4′^I^), 37.8 (C3′), 27.5 (C3), 13.3 (CH_2_I); HR MS [ES^+^]: *m/z* calcd. for C_30_H_26_^79^Br^35^Cl_2_IN_2_ONa 728.9548, found 728.9554 [M+H]^+^.

## 4. Conclusions

The natural product perophoramidine (**1**) continues to challenge synthetic organic chemists. This report describes how the required presence of the two chlorines and one bromine in **1** forced us into a change in synthetic approach compared to our previous reports on dehaloperophoramidine (**2**). The optimization of an NCS-mediated intramolecular C–N bond-forming reaction at the indole 2-position was achieved and led to a suitably halogen-substituted indoloquinoline core structure. Two attempts to progress further towards the structure of perophoramidine (**1**) are also described. In one of these approaches, the presence of the halogens blocked a previously observed undesired reaction pathway. However, an alternative reaction pathway occurred, leading to a major rearrangement of the core structure of the molecule and delivering an interesting furo[2,3-b]indole-containing structure. Throughout this work, small-molecule X-ray crystallographic analysis has proved essential.

## Data Availability

Data is contained within the article or supplementary material.

## Supplementary Materials

Schemes S1–S10, Tables S1–S3, Figures S1–S5 and additional experimental details. CIF files for X-ray structure of **27** and **41**.

## Sample Availability

Samples of the compounds are not available from the authors.
