# Supplementary material for: Synthesis of Indoloquinolines: An Intramolecular Cyclization Leading to Advanced Perophoramidine-Relevant Intermediates
Source: Molecules. 2021 Oct 5;26(19):6039. doi: 10.3390/molecules26196039 (PMC8513048; doi:10.3390/molecules26196039)
Supplement: Supplementary file 1 [file molecules-26-06039-s001.zip › molecules-1400867-supplementary.pdf]

Supplementary Material

Synthesis of indoloquinolines: an intramolecular cyclization leading to advanced Perophoramidine-relevant intermediates

Craig A. Johnston, David B Cordes, Tomas Lebl, Alexandra M. Z. Slawin, Nicholas J. Westwood*

Pages S2-S6	Intermolecular NCS-Mediated Reactions in Model Systems
Pages S7-S10	Optimisation of the NCS-mediated Cyclisation of **9**
Pages S10	Comparison of rate of addition-elimination and Claisen rearrangement with the dehalo system
Page S11	Proposed route to Perophoramidine **1** via an ester alkylation reaction
Pages S12-S14	Outcome of attempted iodetherification reaction of **35**
Pages S14-S18	Additional Experimental Information
Page S19	X-ray Crystallography data
Pages S19-20	References

Intermolecular NCS-Mediated Reactions in Model Systems

Before attempting the intramolecular NCS-mediated cyclisation reaction of **9** (Scheme 5), less complex intermolecular reactions were examined. The reaction of commercially available methyl indole-3-carboxylate (**5**, **Scheme S1**) with N-benzyl-3-bromoaniline (**S1**) was studied. **S1** was proposed as a suitable model substrate due to the appropriately positioned bromo-substituent and N-benzyl protection. Aniline **S1** was synthesised by reacting 3-bromoaniline (**S2**) with benzaldehyde, followed by treatment with sodium triacetoxyborohydride in 69% yield.

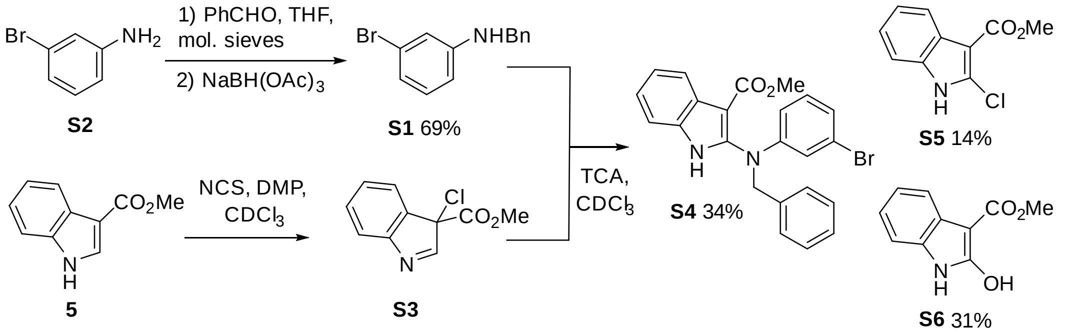

Scheme S1. The synthesis of aniline **S1** was carried out *via* the reductive amination of aniline **S2** with benzaldehyde. NCS-mediated coupling reaction of indole **5** with aniline **S1** proceeded *via* intermediate **S3** before forming three different products. These were found to be the expected tertiary amine product **S4**, 2-chloroindole **S5** and 2-hydroxyindole **S6.**

The NCS-mediated coupling reaction of indole **S1** with **S2** was assessed using ^{1}H NMR analysis. In brief, this enabled monitoring of the formation of the chloroindolenine intermediate **S3** prior to reaction with **S1**. The analysis (**Figure S1A**) found that an excess of NCS was required for the complete conversion of **5** to **S3**. This conversion required 6 hours at room temperature when 2 equivalents of NCS was added. Once intermediate **S3** had been formed, an excess of aniline **S1** was added and after stirring for 18 hrs at rt, the expected product **S4** was isolated in 34% yield (**Scheme S1**). Co-products were identified as **S5** (14%) and **S6** (31%). **S6** was presumably formed by the reaction of intermediate **S3** with water followed by the elimination of HCl. These initial studies suggested that the use of strictly dry conditions may improve this reaction.

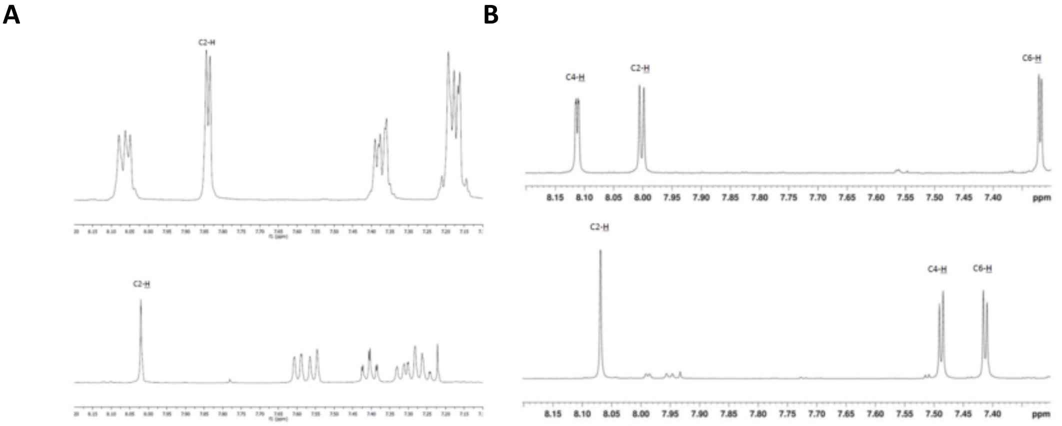

Figure S1. Aromatic region of ^{1}H NMR analysis of: **A.** The starting indole **5** (top spectrum) and the conversion of **5** to chloroindolenine intermediate **S3** (bottom spectrum) after reaction at rt for 6 hrs with NCS (2.0 eq). The signal corresponding to the C2 proton (doublet in **5** due to coupling to the NH; singlet in **S3**) is highlighted. **B.** The starting indole **S7** (top spectrum) and the conversion of **S7** to chloroindolenine intermediate **S9** (bottom) after reaction at rt for 24 hrs with NCS (2.0 eq). Analogous changes to the chemical shift and multiplicity of the signal corresponding to the C2 proton were observed in addition to a dramatic shift in the signal for the C4 proton.

The use of alternative electrophilic halogen sources in place of NCS has been reported in the literature for reactions of this type, however when the reactions with *N*-bromosuccinimide (NBS),[S1] *N*-iodosuccinimide (NIS),[S2] *tert*-butyl hypochlorite[S3] and sodium dichloroisocyanurate[S4] were attempted, **S4** could not be isolated in significant yields.

The potential impact of the chlorine substituents on the formation of the required intermediate was also assessed using dichloroindole **S7** (**Scheme S2**). **S7** was prepared by Pinnick oxidation [S5] of aldehyde **15** to carboxylic acid **S8** which was converted to methyl ester **S7** by refluxing in methanol in the presence of sulfuric acid. The NCS-mediated coupling reaction of **S7** was then followed using ^{1}H NMR analysis (**Figure S1B**). It was observed (as expected) that formation of intermediate **S9** from **S7** (**Scheme S2**) was slower than formation of **S3** from **5** under analogous conditions presumably due to the electron-withdrawing effect of the chlorine substituents. Stirring for 24 hrs at rt with two equivalents of NCS was required for complete conversion of **S7** to **S9** (*c.f.* 6 hrs for complete formation of **S3** from **5**).

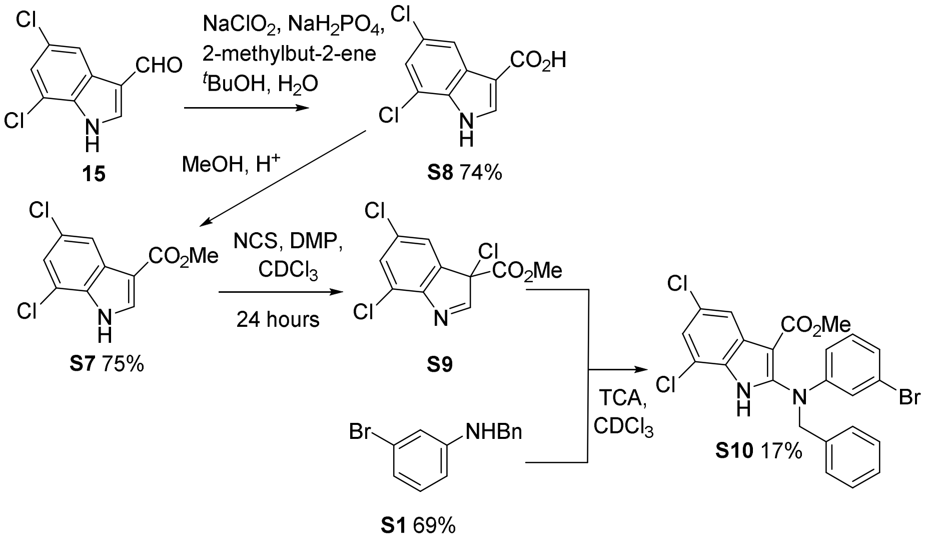

Scheme S2. The synthesis of methyl dichloroindole carboxylate (**S7**) from aldehyde **15** was carried out using a Pinnick oxidation to form acid **S8** in reasonable yield followed by esterification by refluxing in methanol in the presence of sulphuric acid. The NCS-mediated coupling reaction of **S7** proceeded *via* the *in situ* generation of chloroindolenine intermediate **S9** before reaction with aniline **S1** formed 2-substituted indole **S10**.

Following addition of aniline **S1** to the solution of **S9**, the mixture was stirred for an additional 24 hrs at rt before the crude mixture was purified. It was found that the coupled product **S10** (**Scheme S2**) could only be isolated pure in 17% yield (additional amounts of **S10** were present but could not be separated from excess **S1**). Whilst the intermolecular NCS-mediated model cyclisations were far from perfect, it was viewed that as reaction of substrate **9** was an intramolecular process not only would some of the separation challenges experienced with **S10** not occur but the increased effective molarity of the nitrogen nucleophile would aid ring formation. One additional challenge for the intramolecular process involving **9** that was not investigated in these intermolecular model reactions was the selectivity of the NCS-mediated chlorination at the indole 3-position over chlorination in the aniline ring (see manuscript for more discussion).

***N*-Benzyl-3-bromoaniline (S1)[S6]**

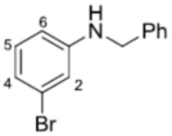

A solution of 3-bromoaniline (**S2**, 11.85 g, 68.90 mmol) and benzaldehyde (7.31 g, 68.90 mmol) in THF (75 mL) was prepared in a flask containing 3Å molecular sieves. After stirring for 2 hours at room temperature, sodium triacetoxyborohydride (21.93 g, 103.5 mmol) was added and the reaction stirred for a further 18 hours before the solvent was removed under reduced pressure. The residue was dissolved in ethyl acetate (50 mL) and washed with 2 M NaOH (2 x 50 mL) before the combined organic extracts were washed with NaCl (aq.) (50 mL) dried ($MgSO_4$) and the solvent removed under reduced pressure. The crude product was purified by column chromatography (10% EtOAc / Hexanes) giving **S2** as a orange-yellow oil (12.40 g, 69%). Spectroscopic data was in accordance with that published in the literature.[S6] **^{1}H NMR** (400 MHz, $CDCl_3$) δ 7.41-7.31 (m, 5H, 5 x ArC-H), 7.05 (t, *J* = 8.0 Hz, 1H, C5-H). 6.87 (ddd, *J* = 7.9, 1.8, 0.8 Hz, 1H, C4-H), 6.82 (t, *J* = 2.1 Hz, 1H, C2-H), 6.57 (ddd, *J* = 8.2, 2.4, 0.8 Hz, 1H, C6-H), 4.34 (s, 2H, CH_2), 4.11 (br. s, 1H, NH); **^{13}C NMR** (100 MHz, $CDCl_3$) δ 149.4 (C1), 138.7 (Ar-C), 130.6 (C5), 128.8 (2 x ArC-H), 127.5 (2 x ArC-H), 126.7 (ArC-H) 123.3 (C3), 120.3 (C4), 115.4 (C2), 111.6 (C6), 48.1 (CH_2); **LR MS [ES$^+$]**: *m/z* calcd. for $C_{13}H_{13}{}^{79}BrN$ 262.02, found 262.13 $[M+H]^+$.

Methyl 2-(benzyl(3-bromophenyl)amino)-1*H*-indole-3-carboxylate (S4)

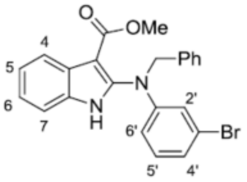

To a solution of **5** (87 mg, 0.50 mmol) in $CDCl_3$ (5 mL) was added NCS (146 mg, 1.10 mmol) followed by DMP (45 mg, 0.28 mmol). The reaction was stirred at room temperature for 6 hours before a solution of aniline **S1** (262 mg, 1.00 mmol) and TCA (20 mg, 0.13 mmol) in $CDCl_3$ (5 mL) was added. The solution was stirred for 48 hours before a saturated solution of $NaHCO_3$ (aq.) (20 mL) was added and the mixture extracted with DCM (3 x 10 mL). The combined organic extracts were washed with 1 M HCl (aq.) (20 mL). The organic phase was then dried ($MgSO_4$), filtered and the solvent removed under reduced pressure. The crude product was purified by column chromatography (5-25% EtOAc / Hexanes) giving 3 products: **S4** as a pale yellow solid (74 mg, 34%). **m.p.** 54-57 °C; **I.R.** (KBr) ν_{max} 3261, 1590, 1543, 1476, 1452, 1211, 1115, 722 cm^{-1}; **^{1}H NMR** (400 MHz, $CDCl_3$) δ 8.28 (br. s, 1H, NH), 8.10-8.05 (m, 1H, C-H), 7.28-7.16 (m, 8H, 8 x C-H), 6.95-6.88 (m, 2H, 2 x C-H), 6.84 (dd, *J* = 1.9, 2.3 Hz, 1H, C-H), 6.62 (ddd, *J* = 8.1, 2.4, 1.2 Hz, 1H, C-H), 4.97 (s, 2H, CH_2), 3.75 (s, 3H, CH_3); **^{13}C NMR** (100 MHz, $CDCl_3$) δ 164.4 (C=O), 148.8 (C), 145.5 (C), 138.0 (C), 132.0 (C), 130.4 (CH), 128.9 (2 x CH), 127.5 (CH), 126.8 (2 x CH), 126.3 (C), 123.3 (CH), 123.2 (C), 123.1 (CH), 122.2 (CH), 121.9 (CH), 118.5 (CH), 114.2 (CH), 111.0 (CH), 100.0 (C), 56.6 (CH_2), 51.0 (CH_3); **LR MS [ES$^-$]**: *m/z* 432.99 $[M-H]^-$.

Methyl 2-chloro-1*H*-indole-3-carboxylate (S5)[S7]

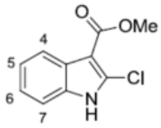

Obtained during the synthesis of **S4** as a white solid (15 mg, 14%). Spectroscopic data was in accordance with that published in the literature.[S7] **m.p.** 181-183 °C (lit.[S7] 183 °C); **^{1}H NMR** (400

MHz, acetone-d6) δ 11.55 (br. s, 1H, NH) 8.07 (dd, *J* = 6.8, 2.0 Hz, 1H), 7.43 (dd, *J* = 7.0, 1.8 Hz, 1H), 7.27-7.20 (m, 2H), 3.90 (s, 3H, CH_3); **^{13}C NMR** (101 MHz, acetone-d6) δ 164.4 (C=O), 135.3 (C7a), 130.6 (C2), 127.2 (C3a), 124.0, 122.7, 121.8, 111.9, 104.3 (C3), 51.1 (CH_3); **LR MS [ES$^-$]**: *m/z* 208.07 $[M-H]^-$.

Methyl 2-hydroxy-1*H*-indole-3-carboxylate (S7) [S8]
]

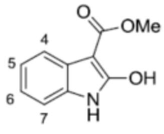

Obtained during the synthesis of **S4** as a white solid (28.9 mg, 31%). **m.p.** 86-88 °C; **I.R.** (KBr) ν_{max} 3165, 3099, 1764, 1622, 1230, 753 cm^{-1}; **^{1}H NMR** (400 MHz, $CDCl_3$) δ 8.66 (br. s, 1H, NH), 7.43 (d, *J* = 7.6 Hz, 1H), 7.35 (td, *J* = 7.8, 1.2 Hz, 1H), 7.12 (td, *J* = 7.6, 1.0 Hz, 1H), 6.97 (d, *J* = 7.9 Hz, 1H), 3.82 (s, 3H, CH_3); **^{13}C NMR** (100 MHz, DMSO-d6) δ 170.2 (C=O), 165.2 (C2), 142.0 (C7a), 131.6, 126.6 (C3a), 124.6, 123.1, 110.9, 65.02 (C3), 54.3 (CH_3); **LR MS [ES$^+$]**: *m/z* 190.05 $[M-H]^+$.

5,7-Dichloroindole-3-carbaldehyde (15)[S9]

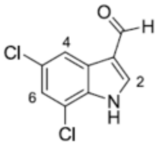

Phosphorus oxychloride (8.65 mL, 92.8 mmol) was added slowly to DMF (18 mL) at 5-10 °C before the mixture was allowed to warm to room temperature over 30 minutes. A solution of **13** (6.00 g, 32.2 mmol) in DMF (8 mL) was added dropwise and the solution stirred at 35 °C for 1 hour before being poured into ice-water (100 mL). A 20% aqueous solution of sodium hydroxide (125 mL) was added dropwise until 20 mL had been added then the remainder was added in one portion. The mixture was then refluxed for 10 minutes before cooling to room temperature and the precipitate formed was collected by filtration and washed with cold water (50 mL) before being recrystallized from 50:50 EtOAc : hexanes to give **15** as a white solid (5.99 g, 87%). Spectroscopic data was in accordance with that published in the literature.[S9] **m.p.** 226-228 °C (lit.[S9] 227-230 °C); **^{1}H NMR** (400 MHz, d^6-DMSO) δ 12.77 (br. s, 1H, N-H), 9.95 (s, 1H, CHO), 8.45 (s, 1H, C2-H), 8.03 (d, *J* = 1.8 Hz, 1H, C4- H), 7.48 (d, *J* = 1.8 Hz, 1H, C6-H); **^{13}C NMR** (100 MHz, d^6-DMSO) δ 185.2 (CHO), 140.2 (C2), 132.9 (C7a), 126.8 (C5), 126.5 (C3a), 122.6 (C6), 118.9 (C4), 118.2 (C7), 117.7 (C3); **LR MS [ES$^-$]**: *m/z* 211.98 $[M-H]^-$.

5,7-Dichloroindole-3-carboxylic acid (S8)

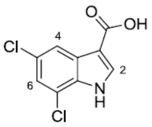

A solution of **15** (3.00 g, 14.85 mmol) in *t*BuOH : H_2O (1:1, 85 mL) was prepared and a solution of 2-methylbut-2-ene in THF (2 M, 16.20 mL, 32.37 mmol) was added followed by $NaClO_2$ (17.40 g, 192.30 mmol) and NaH_2PO_4 (25.70 g, 184.50 mmol). The reaction was stirred overnight before being diluted with water (100 mL) and extracted with ethyl acetate (2 x 75 mL). The combined organic layers were dried ($MgSO_4$), filtered and the solvent removed under reduced pressure. The crude product was recrystallised from chloroform to give **S8** as an orange-brown solid (2.39 g, 74%). **m.p.** 280-282 °C; **I.R.** (KBr) ν_{max} 3356, 3133, 1703, 1460, 1179 cm^{-1}; **^{1}H NMR** (400 MHz, MeOD) δ 8.03 (s, 1H, C2-H), 8.01 (d, *J* = 1.9 Hz, 1H, C4-H), 7.27 (d, *J* = 1.9 Hz, 1H, C6-H); **^{13}C NMR** (100 MHz, MeOD) δ 167.9 (C=O), 135.2

(C2), 133.8 (C7a), 129.8 (C3a), 128.3 (C5), 123.0 (C6), 120.5 (C4), 119.0 (C7), 110.0 (C3); **LR MS [ES$^-$]**: *m/z* 227.92 [M-H]$^-$.

5,7-Dichloroindole-3-carboxylate (S7)

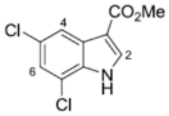

A solution of **S8** (2.30 g, 9.42 mmol) in methanol (100 mL) was prepared and conc. sulphuric acid (2 mL) was added. The mixture was heated to reflux for 18 hours before being concentrated under reduced pressure. Water (25 mL) was added and the solution was neutralised with NaOH. The ester was then extracted with ethyl acetate (25 mL) and washed with NaCl (aq.) (15 mL) before being dried ($MgSO_4$) and the solvent removed under reduced pressure. **S7** was obtained as a white solid (1.84 g, 75%). **m.p.** 202-204 °C; **I.R.** (KBr) ν_{max} 3304, 1692, 1631, 1446, 1173 cm^{-1}; **^{1}H NMR** (400 MHz, $CDCl_3$) δ 8.76 (br. s, 1H, NH), 8.11 (d, *J* = 1.8 Hz, 1H, C4-H), 8.00 (d, *J* = 2.9 Hz, 1H, C2-H), 7.32 (d, *J* = 1.8 Hz, 1H, C6-H), 3.96 (s, 3H, CH_3); **^{13}C NMR** (100 MHz, $CDCl_3$) δ 164.4 (C=O), 132.1 (C2), 132.0 (C), 128.0 (C), 127.6 (C), 122.9 (C6), 120.0 (C4), 119.8 (C), 110.3 (C), 51.4 (CH_3). **LR MS [ES$^-$]**: *m/z* 242.00 [M-H]$^-$.

Methyl 2-(benzyl(3-bromophenyl)amino)-5,7-dichloro-1*H*-indole-3-carboxylate (S10)

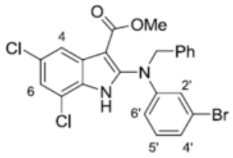

To a suspension of **S7** (50 mg, 0.205 mmol) in $CDCl_3$ (2.2 mL) in the presence of 3Å molecular sieves was added NCS (68 mg, 0.512 mmol) and DMP (13 mg, 0.115 mmol). The mixture was stirred at room temperature for 24 hours before a solution of **S1** (107 mg, 0.410 mmol) and TCA (8 mg, 0.049 mmol) in $CDCl_3$ (2.2 mL) was added. The mixture was stirred for a further 24 hours at room temperature before the solvent was removed under reduced pressure and the crude product purified by column chromatography (0-20% EtOAc / Hexanes). **S10** was obtained as a yellow solid (18 mg, 17%). **^{1}H NMR** (400 MHz, $CDCl_3$) δ 8.23 (br. s, 1H, NH), 7.94 (d, *J* = 1.9 Hz, 1H, C-H), 7.29-7.25 (m, 5H, 5 x C-H), 7.17 (d, *J* = 1.8 Hz, 1H, C-H), 7.01-6.97 (m, 2H, 2 x C-H), 6.93-6.92 (m, 1H, C-H), 6.72- 6.68 (m, 1H, C-H), 5.00 (s, 2H, CH_2), 3.75 (s, 3H, CH_3). **LR MS [ES$^-$]**: *m/z* 501.03 [M-H]$^-$.

Optimisation of the NCS-mediated Cyclisation of 9

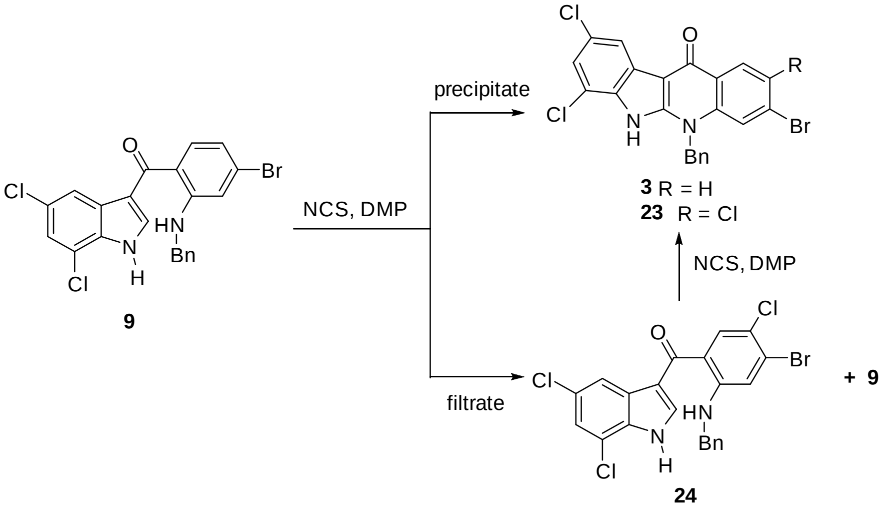

Scheme S3. Preliminary attempts at NCS-mediated cyclisation of **9** led to formation of a precipitate that contained the desired cyclized product **3** and a second product assigned as **23**. Significant optimisation of this reaction was required. NCS = N-chlorosuccinimide; DMP = N,N'-dimethylpiperazine.

The initial reaction was carried out using NCS (1.0 equiv.) and DMP (0.5 equiv.) in DCM at room temperature. As described in Scheme S3 (Scheme 5 in manuscript and the associated text), this led to the formation of a small amount of precipitate. This precipitate was poorly soluble in most organic solvents but some of the material dissolved in d6-DMSO enabling NMR analysis (Figures S2 and S3). Comparison with the NMR analysis of a purified sample of **3** confirmed its presence as the minor product (Figure S2 legend). Assignment of the second compound in the precipitate relied on its synthesis from a pure sample of the chlorinated precursor **24** (Scheme S3, Figure S3 legend).

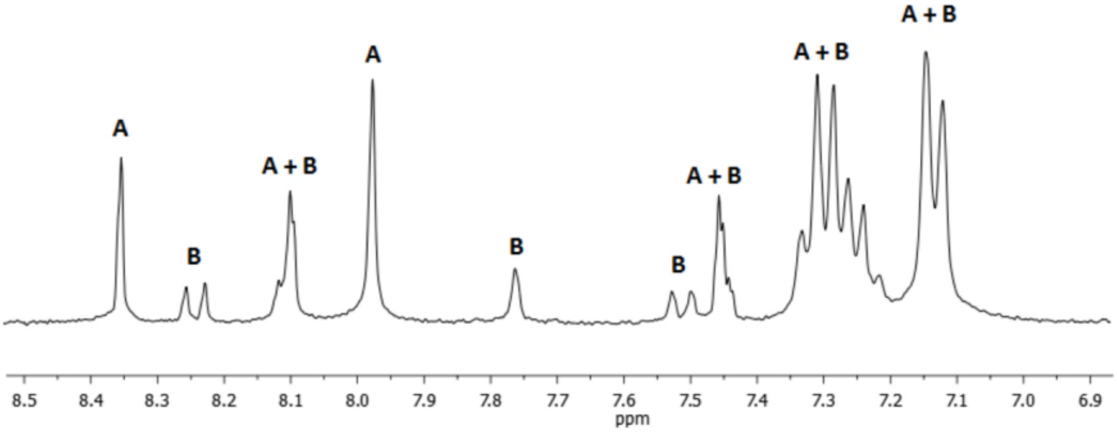

Figure S2. The aromatic region of the ^1H NMR spectrum (DMSO-d_6) of the precipitate formed in the reaction of **9** with NCS indicated that two different compounds were present (labelled A for the chlorinated analogue **23** and B for **3**). The assigned NMR spectrum for **3** was as follows: ^1H NMR (400 MHz, d6-DMSO) δ 12.45 (br. s, 1H, NH), 8.27 (d, *J* = 8.5 Hz, 1H, C1-H), 8.14 (d, *J* = 1.9 Hz, 1H, C10-H), 7.78 (d, *J* = 1.6 Hz, 1H, C4-H), 7.54 (dd, *J* = 8.5, 1.6 Hz, 1H, C2-H), 7.46 (d, *J* = 1.9 Hz, 1H, C8-H), 7.36-7.26 (m, 3H, 3 x ArC-H), 7.18-7.16 (m, 2H, 2 x ArC-H), 5.98 (s, 2H, CH_2).

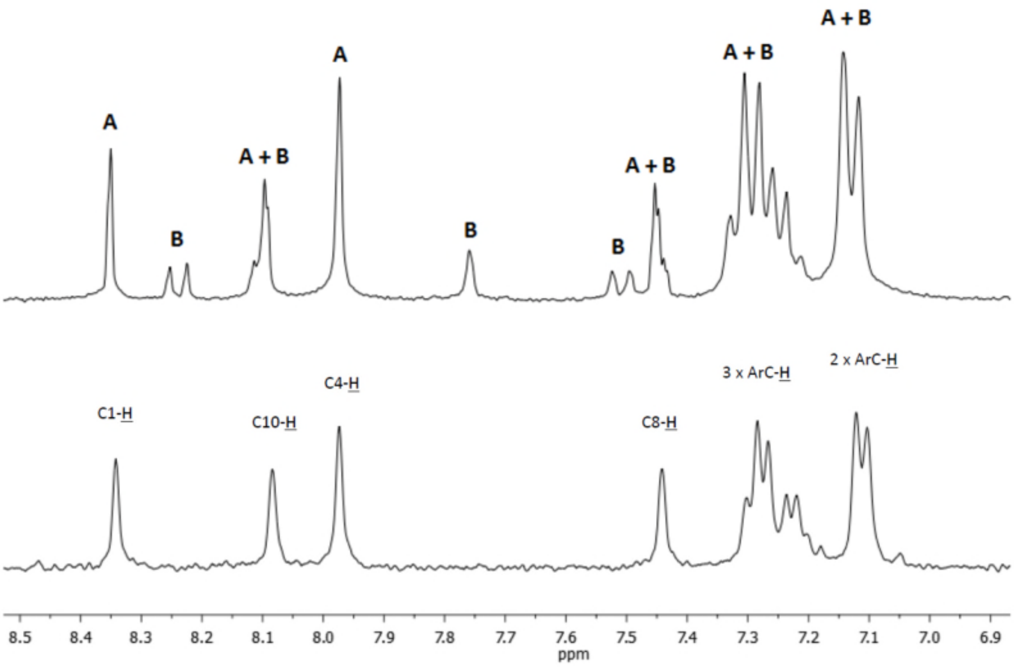

Figure S3. The aromatic region of the ^{1}H NMR spectrum of **23** (bottom) was found to be consistent with product A from the reaction of **9** with NCS, confirming **23** as the chlorinated analogue of **3** (Scheme S3). The assigned NMR spectrum for **23** was as follows: ^{1}H NMR (400 MHz, d6-DMSO) δ 12.50 (br. s, NH), 8.37 (s, 1H, C1-H), 8.12 (d, *J* = 1.9 Hz, 1H C10-H), 7.99 (s, 1H, C4-H), 7.47 (d, *J* = 1.9 Hz, 1H, C8-H), 7.37-7.26 (m, 3H, 3 x ArC-H), 7.18-7.15 (m, 2H, 2 x ArC-H), 5.98 (s, 2H, CH_2).

Subsequent studies looked to optimise the solvent and base used in the reaction (Tables S1 and S2 and experimental procedures below).

Table S1. The results of a solvent screen for the cyclisation of **9**.

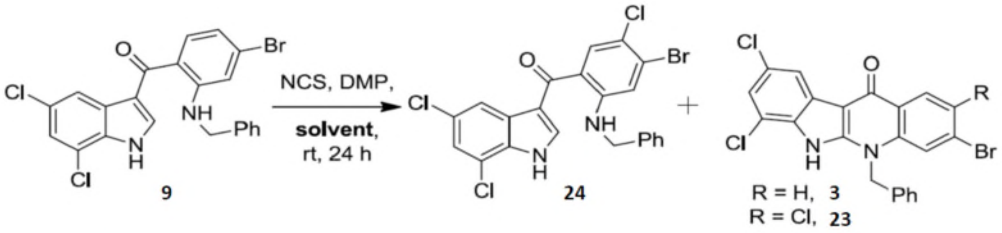

Entry	Solvent	Precipitate Isolated[a]	Ratio of 9 : 24 in Filtrate[b]
1	DCM	6 mg	60:40
2	Methanol	-[c]	40:60
3	Acetone	3 mg	61:39
4	THF	2 mg	64:36
5	Acetonitrile	8 mg	80:20
6	Hexane	-[c]	67:33
7	Toluene	4 mg	49:51

[a] A mixture of the required product **3** and the over-chlorinated product **23** were obtained as an inseparable mixture in the form of a precipitate; [b] The filtrate contained a mixture of unreacted starting material **9** and chlorinated aniline product **24**; [c] no precipitate isolated.

Table 2. The results of a screen of 6 different bases for the cyclisation of **9** in MeCN.

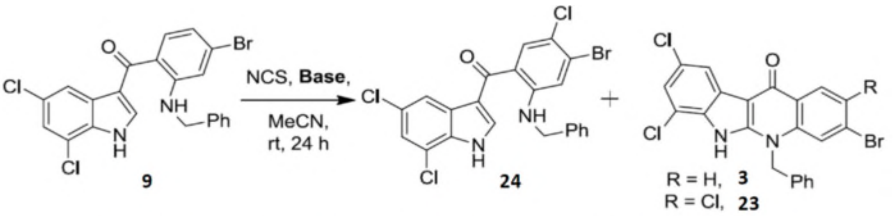

Entry	Base	Precipitate Isolated (mg)	Ratio of 3 : 23 in Precipitate	Ratio of 9 : 24 in Filtrate
1	DMP	12	100 : 0	90 : 10
2	NaH	10	0 : 100	-[b]
3	Et_3N	24	100 : 0	85 : 15
4	DMAP	5	0 : 0[a]	-[b]
5	DIPEA	5	100 : 0	90 : 10
6	Pyridine	-[c]	-[c]	-[b]

[a] Isolated precipitate neither **3** nor **23**; [b] no **9** observed in filtrate, mixtures of **24** and other unidentified products observed; [c] no precipitate obtained.

Experimental protocols used in the solvent and base screening experiments used to optimize the preparation of 5-Benzyl-3-bromo-7,9-dichloro-5*H*-indolo[2,3-*b*]quinolin-11(6*H*)-one (3)

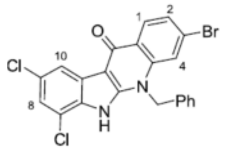

Method used in solvent screen: To a solution of **9** (50 mg, 0.105 mmol) in the solvent under test (1.2 mL) was added *N*-chlorosuccinimide (14 mg, 0.105 mmol) and *N*,*N'*dimethylpiperazine (8 µl, 0.060 mmol). The mixture was stirred for 24 hours at room temperature and then any precipitate that had formed was collected by filtration, washed with DCM and dried under vacuum (mass of dried precipitate recorded in Table S1). The solvent was removed from the filtrate under reduced pressure before analysis of the crude filtrate was carried out by ^{1}H NMR with the ratio of the two main compounds present in the filtrate recorded in Table S1.

Method used in base screen: To a solution of **9** (50 mg, 0.105 mmol) in acetonitrile (1.2 mL) was added *N*-chlorosuccinimide (14 mg, 0.105 mmol) and the base under test (0.126 mmol). The mixture was stirred for 24 hours at room temperature before any precipitate formed was collected by filtration, washed with DCM and dried under vacuum (mass of dried precipitate recorded in Table S2). The solvent was removed from the filtrate under reduced pressure which was then analysed by ^{1}H NMR with the ratio of the two main compounds present in the filtrate recorded in Table S2.

Small scale preparation of 5-Benzyl-3-bromo-2,7,9-trichloro-5*H*-indolo[2,3-*b*]quinolin-11(6*H*)-one (23)

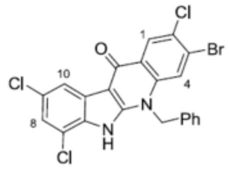

To a solution of **24** (14 mg, 0.027 mmol) in DCM (0.4 mL) was added NCS (4 mg, 0.027 mmol) followed by DMP (2 µL, 0.015 mmol) and the mixture stirred for 24 hours at room temperature. The precipitate formed was then collected by filtration and washed with DCM giving **23** as a white solid (6 mg, 43%). ^{1}H NMR (400 MHz, d6-DMSO) δ 12.50 (br. s, NH), 8.37 (s, 1H, C1-H), 8.12 (d, *J* = 1.9 Hz, 1H C10-H), 7.99 (s, 1H, C4-H), 7.47 (d, *J* = 1.9 Hz, 1H, C8-H), 7.37-7.26 (m, 3H, 3 x ArC-H), 7.18-7.15 (m, 2H, 2 x ArC-H), 5.98 (s, 2H, CH_2); LR MS [ES$^-$]: *m/z* 502.82 [M-H]$^-$.

Isolation of (2-(Benzylamino)-4-bromo-5-chlorophenyl)(5,7-dichloro-1H-indol-3-yl)methanone (24) from reaction of 9 with NCS

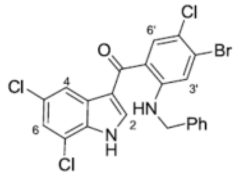

To a solution of **9** (128 mg, 0.27 mmol) in $CDCl_3$ (3 mL) was added *N*-chlorosuccinimide (36 mg, 0.54 mmol) and *N*,*N*'dimethylpiperazine (17 mg, 0.15 mmol). The mixture was stirred for 24 hours at room temperature before the precipitate was collected by filtration. The precipitate was found to contain a mixture of both **2** and **23** which could not be separated. The filtrate was concentrated *in vacuo* and purified by column chromatography (5-25% EtOAc / Hexanes) giving **24** as a yellow solid (14 mg, 10%). m.p. 50-52 °C; I.R. (KBr) ν_{max} 3409, 1705, 1603, 1420, 1177 cm^{-1}; ^{1}H NMR (400 MHz, $CDCl_3$) δ 8.88 (br. s, 1H, NH), 8.09 (d, *J* = 1.7 Hz, 1H, C4-H), 8.05 (br. s, NH-Bn), 7.69 (s, 1H, C6'-H), 7.60 (d, *J* = 2.8 Hz, 1H, C2-H), 7.31 – 7.17 (m, 6H, 5 x Ph-H, C6-H), 6.95 (s, 1H, C3'-H), 4.34 (s, 2H, CH_2); ^{13}C NMR (100 MHz, $CDCl_3$) δ 190.4 (C=O), 149.0 (C2'), 137.6 (ArC), 133.1 (C6'), 132.8 (C2), 132.2 (C7a), 128.9 (ArC), 128.7 (ArCH), 128.6 (C3), 128.3 (ArC), 127.6 (ArCH), 127.3 (ArCH), 123.8 (C6), 120.6 (C4), 120.4 (ArC), 119.5 (ArC), 118.1 (ArC), 117.3 (ArC), 116.7 (C3'), 47.3 (CH_2); HR MS [ES$^-$]: *m/z* calcd. for $C_{22}H_{14}{}^{79}Br^{35}Cl_3N_2O$ 504.9277, found 504.9280 [M-H]$^-$.

Comparison of rate of addition-elimination and Claisen rearrangement with the dehalo system

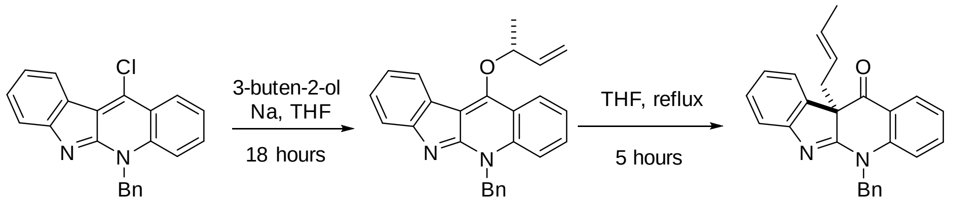

Scheme S4. The previously reported [S10] model system reaction of chloride **S11** with the alkoxide formed from the reaction of 3-buten-2-ol with sodium yielded ether **S12** which was rearranged to ketone **S13** in refluxing THF. Both of these steps required considerably longer reactions times than the corresponding reactions in the halogenated series shown in Scheme 6 of the manuscript.

Proposed route to Perophoramidine 1 via an ester alkylation reaction

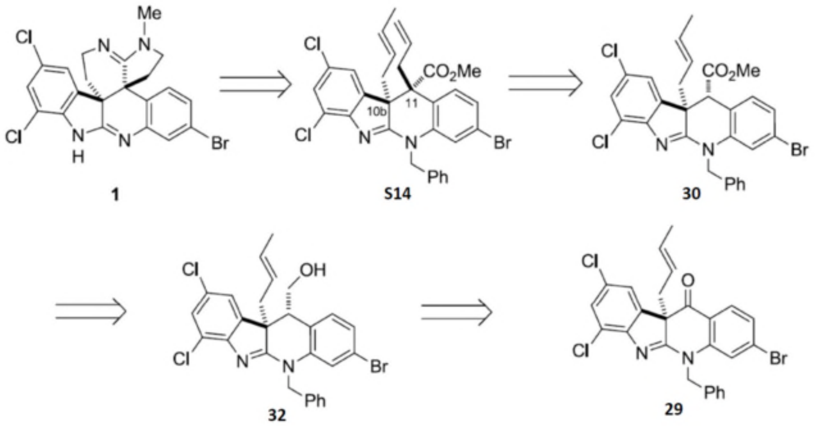

Scheme S5. One possible retrosynthesis of perophoramidine **1** in which the C11 quaternary centre could be installed by oxidation of alcohol in **32** followed by esterification formation to give **30**. Deprotonation at the C11 position and quenching with allyl bromide could form the C-11 allyl group of **S14** and **1**.

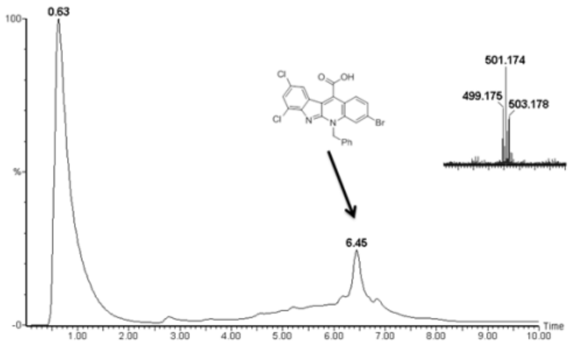

Figure S4. LC-MS analysis of the crude reaction mixture obtained on oxidation of **32** after 5 hours. The main species present in the mixture was assigned as compound **34** (structure shown). The mass spectrum associated with the main peak had m/z = 501.17 $[M+H]^+$ consistent with structure **34** ($C_{23}H_{13}{}^{81}Br^{35}Cl_2N_2O_2$ expected $[M+H]^+$ = 500.96). The peak at 0.63 minutes retention time is the solvent front and no relevant m/z signals were identified from it.

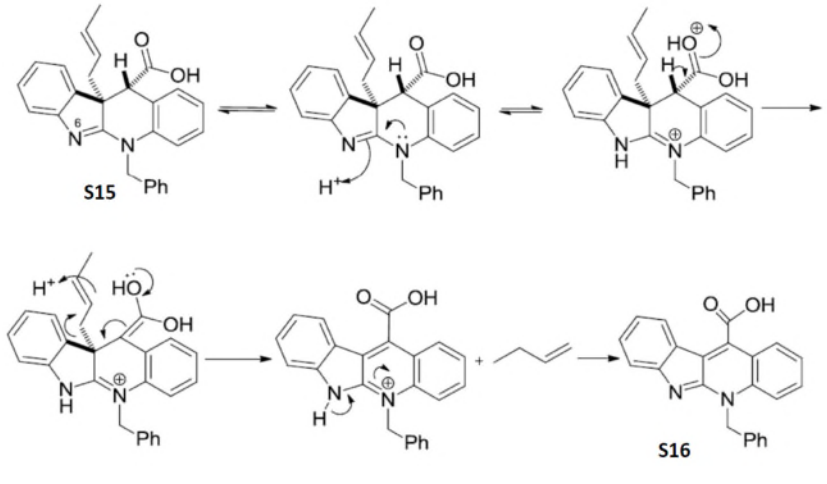

Scheme S6. One possible mechanism for the conversion of the initial product acid **S15** of the Jones oxidation in the crotyl dehalo-series to the corresponding acid **S16**. This mechanism may be relevant to the conversion of **32** to **34** via **33** in this work (Scheme 7 in manuscript).

Outcome of attempted iodetherification reaction of 35

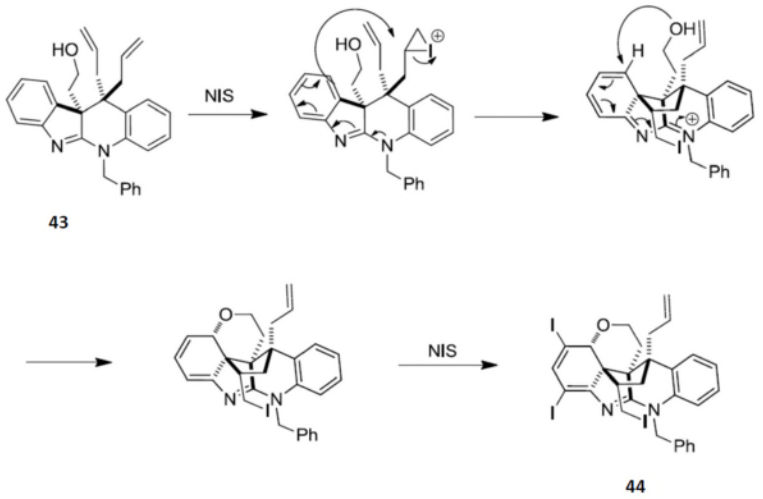

Scheme S7. One possible mechanism for the previously reported conversion of diallyl-containing **43** to the complex polycyclic system **44** on treatment with excess NIS [S11]. It was initially hoped that an iodoetherification reaction of **43** might occur in this reaction.

Compound **45** was isolated as a single diastereomer and represented the major compound in the crude reaction mixture. The structure of **45** was tentatively assigned based on analysis of the NMR data including some diagnostic correlations observed in the HMBC spectrum. These included the multiple bond correlation between the peaks corresponding to the N-benzyl C<u>H</u>$_2$ group (4.63 and 4.46 ppm) and C2' at 56.9 ppm (Figure S5 and Scheme S8). These two atoms (the NC<u>H</u>$_2$ and <u>C</u>2') would be 7 bonds away from each other if **36** had been formed and no correlation would have been expected. It was also noted that the correlation between the NC<u>H</u>$_2$ protons and the amidine carbon atom (173.9 ppm in **275**) was not observed. These correlations were diagnostic throughout these studies in showing that the N5-C5a was intact. It therefore appeared that the N5-C5a bond in **35** had been broken and a new bond had been formed between N1' and C2' in **45**.

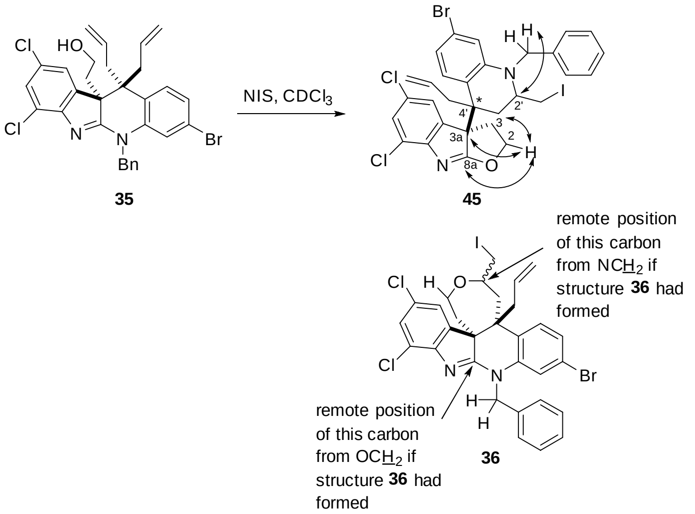

Scheme S8. Structures of desired product **36** and of the product that was actually isolated **45**. Key HMBC correlations shown in Figure S5 are highlighted in **45** as well as the clear inconsistency that would have existed with the observed data if the product had been assigned as **36**.

Another important correlation was observed from the signals relating to C2-H_2 (one of these signals is shown at 4.31 ppm). HMBC correlations were observed to C3 (27.5 ppm), C3a (64.9 ppm) and C8a (193.6 ppm). Whilst the first two of these would be expected for the formation of **36** (Scheme S8), the third correlation would be four bonds away and unlikely to be observed by HMBC. This correlation provided evidence in support of the formation of the O1-C8a bond in **45**.

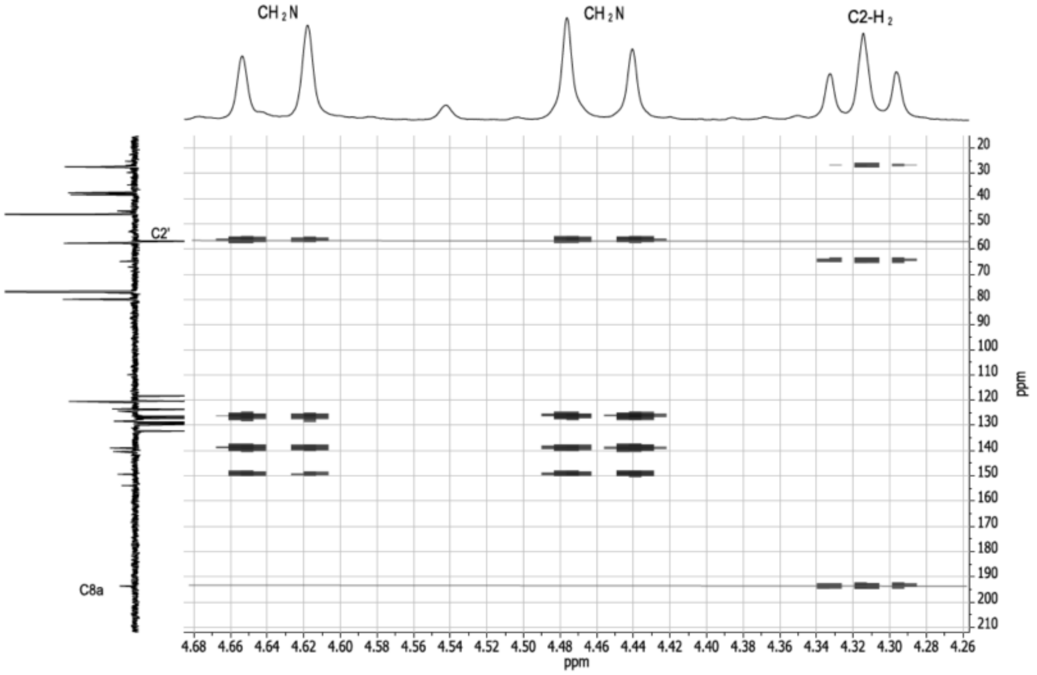

Figure S5. The region of the HMBC spectrum of **45** highlighting the correlations between the C<u>H</u>$_2$N protons and <u>C</u>2' carbon (56.9 ppm). A second important correlation is shown between one of the C2-<u>H</u>$_2$ protons and <u>C</u>8a (173.9 ppm).

More evidence for the formation of **45** was obtained from low resolution mass spectrometric analysis. It was found that as well as the peaks corresponding to the expected $[M+H]^+$ of **45** (expected for $C_{30}H_{27}{}^{79}Br^{35}Cl_2IN_2O$: 706.97; found 706.93), a smaller fragmentation peak was also observed. Upon increasing the cone voltage to increase the degree of fragmentation, it was found that peaks at m/z = 479.95 and 481.95 were obtained. It was proposed that these peaks correspond to cation **S17** (expected $[M+H]^+$ 479.98, 481.98) which may have been formed by breaking the C3a- C4' bond in **45** (Scheme S8). The two peaks of approximately equal intensity were caused by the presence of bromine isotopes ^{79}Br and ^{81}Br (51:49 relative abundance).

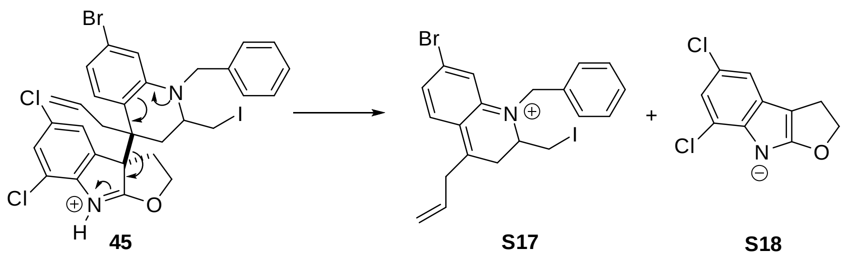

45
Expected $[M+H]^+$ 706.97
found 706.93

S17
Expected M^+ 479.98, 481.98
found 479.95, 481.95

S18
not seen

Scheme S9 – Fragmentation under mass spectrometry ionisation conditions (ES$^+$) of **45** was observed forming observed cation **S17** and presumably **S18** (not observed).

One possible transformation for the conversion of **35** to **45**.

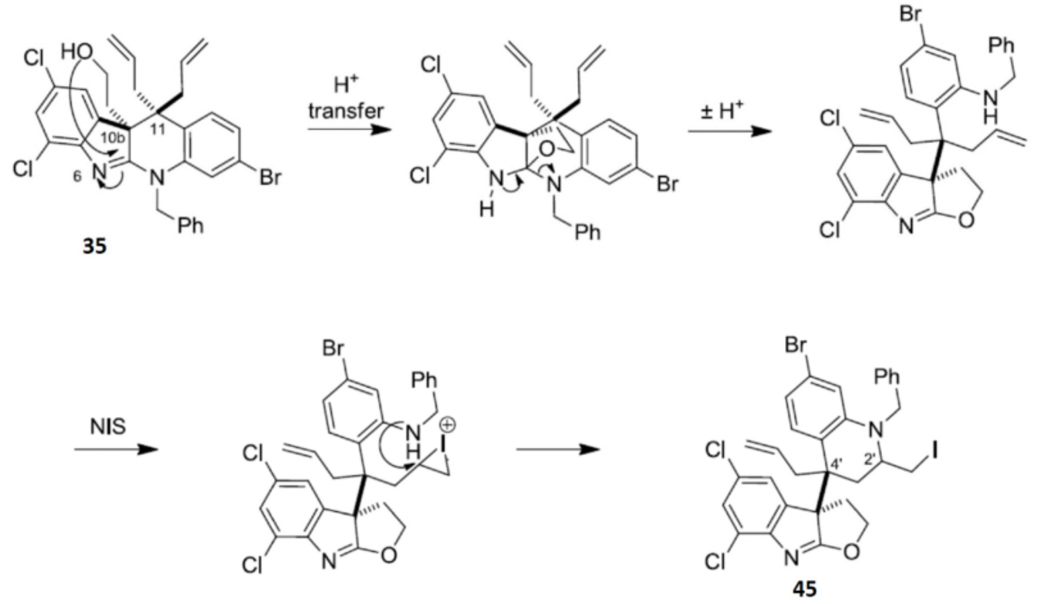

Scheme S10. It was proposed that **35** could be formed by cyclisation of the primary alcohol onto the amidine carbon before ring opening, iodonium ion formation and finally opening of the iodonium ion with the neighbouring nitrogen.

Additional Experimental Information

General Procedures

All chemical and reagents were obtained from either Sigma-Aldrich, Lancaster or Alfa-Aesar and were used as received unless otherwise stated. *N*-Chlorosuccinimide was purified by recrystallisation from the minimum amount of boiling water followed by drying in a vacuum oven. *p*-Toluenesulfonyl chloride was purified by recrystallisation from the minimum amout of boiling petroleum ether. *p*-Toluenesulfonic acid was recrystallized from the minimum amount of boiling ethyl acetate before drying in a vacuum oven. Sodium cyanoborohydride was purified by dissolving in THF (20% w/v), filtering and treating the filtrate with a fourfold volume of DCM before collecting the precipitate by filtration and drying in a vacuum oven. All reactions were carried out under a positive pressure of nitrogen. Tetrahydrofuran (THF), dichloromethane (DCM) and toluene were obtained dry from a solvent purification system (MBraun, SPS-800). All other solvents used were dried by distillation and were stored over 4 Å molecular sieves.

Thin-layer chromatography was performed using glass plates coated with silica gel (with fluorescent indicator UV_{254}). Developed plates were air-dried and analysed under a UV lamp (254/365 nm). Column chromatography was performed using silica gel (40-63 µm, Fluorochem) using either a glass column or a Biotage SP1 / SP4 flash purification system.

5,7-Dichloroindole **(13)[S12]**

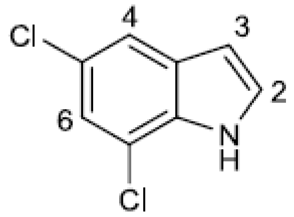

2,4-Dichloronitrobenzene (**14**, 19.20 g, 100 mmol) was dissolved in THF (200 mL) and cooled to -78 °C before a 1.6 M solution of vinylmagnesium chloride in THF (200 mL, 320 mmol) was added dropwise. The reaction was stirred for a further 1 hour at -78 °C before being quenched by the addition of NH_4Cl (aq.) (300 mL). After warming to room temperature, the mixture was extracted with diethyl ether (2 x 200 mL) and the organic extracts were washed with NaCl (aq.) (150 mL), dried ($MgSO_4$), filtered and the solvent evaporated at reduced pressure. The crude product was purified by column chromatography (10% EtOAc / Hexanes) to afford **13** as an orange-brown solid (8.95 g, 48%). Spectroscopic data was in accordance with that published in the literature.[S12] **m.p.** 55-56 °C (lit.[S12] 55-56 °C); **^{1}H NMR** (400 MHz, $CDCl_3$) δ 8.41 (br. s, 1H, N-H), 7.55 (d, *J* = 1.7 Hz, 1H, C4-H), 7.31 (d, *J* = 5.6 Hz, 1H, C2-H), 7.23 (d, *J* = 1.7 Hz, 1H, C6-H), 6.57 (d, *J* = 5.5 Hz, 1H, C3-H); **^{13}C NMR** (100 MHz, $CDCl_3$) δ 131.8 (C7a), 129.7 (C3a), 126.1 (C2), 125.5 (C5), 121.5 (C6), 119.0, (C4), 116.9 (C7), 103.5 (C3); **LR MS [ES$^-$]**: *m/z* 184.01 [M-H]$^-$.

5,7-Dichloroindole-3-carbaldehyde (15)[S9]

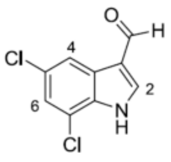

Phosphorus oxychloride (8.65 mL, 92.8 mmol) was added slowly to DMF (18 mL) at 5-10 °C before the mixture was allowed to warm to room temperature over 30 minutes. A solution of **13** (6.00 g, 32.2 mmol) in DMF (8 mL) was added dropwise and the solution stirred at 35 °C for 1 hour before being poured into ice-water (100 mL). A 20% aqueous solution of sodium hydroxide (125 mL) was added dropwise until 20 mL had been added then the remainder was added in one portion. The mixture was then refluxed for 10 minutes before cooling to room temperature and the precipitate formed was collected by filtration and washed with cold water (50 mL) before being recrystallized from 50:50 EtOAc : hexanes to give **15** as a white solid (5.99 g, 87%). Spectroscopic data was in accordance with that published in the literature [S9]. m.p. 226-228 °C (lit. [S9] 227-230 °C). ^{1}H NMR (400 MHz, DMSO) δ 12.77 (br. s, 1H, N-H), 9.95 (s, 1H, CHO), 8.45 (s, 1H, C2-H), 8.03 (d, *J* = 1.8 Hz, 1H, C4-H), 7.48 (d, *J* = 1.8 Hz, 1H, C6-H). ^{13}C NMR (100 MHz, DMSO) δ 185.2 (CHO), 140.2 (C2), 132.9 (C7a), 126.8 (C5), 126.5 (C3a), 122.6 (C6), 118.9 (C4), 118.2 (C7), 117.7 (C3). LR MS [ES$^-$]: *m/z* 211.98 [M-H]$^-$.

4-Bromo-1-iodo-2-nitrobenzene (17)[S13]

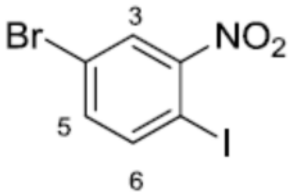

Boron trifluoride diethyl etherate (19.90 mL, 157.06 mmol) was cooled to -30 °C before a solution of **18** (8.10 g, 37.34 mmol) in THF (100 mL) was added dropwise followed by ^{t}butylnitrite (16.34 g, 137.44 mmol) in THF (100 mL). The mixture was allowed to warm to -5 °C before diethyl ether (200 mL) was added and the mixture stirred for a further 10 minutes at -5 °C. The pale yellow precipitate was isolated by filtration and washed with cold diethyl ether (2 x 50 mL) before being added to a solution of potassium iodide (8.80 g, 53.06 mmol) and iodine (6.74 g, 26.54 mmol) in acetonitrile (150 mL). The

solution was stirred at room temperature for 15 minutes before $Na_2S_2O_3$ (aq.) (300 mL) and DCM (300 mL) were added. The mixture was stirred for 5 minutes before the layers were separated and the organic phase dried ($MgSO_4$), filtered and the solvent removed under reduced pressure. **17** was obtained as a yellow solid (10.15 g, 83%) that required no further purification. Spectroscopic data was in accordance with that published in the literature.[S13] **m.p.** 88-89 °C (lit.[S13] 87-89 °C); ^{1}H NMR (400 MHz, $CDCl_3$) δ 8.02 (d, *J* = 2.2 Hz, 1H, C3-H), 7.92 (d, *J* = 8.5 Hz, 1H,C6-H), 7.43 (dd, *J* = 8.5, 2.2 Hz, 1H, C5-H); ^{13}C NMR (75 MHz, $CDCl_3$) δ 154.1 (C2), 143.3 (C6), 136.9 (C5), 128.9 (C3), 123.1 (C4), 84.8 (C1); LR MS [ES$^-$]: *m/z* calcd. for $C_6H_2{}^{79}BrINO_2$ 325.83, found 325.81 [M-H]$^-$.

Synthesis of (2-(Benzylamino)-4-bromophenyl)(5,7-dichloro-1*H*-indol-3-yl)methanone (9) from 21

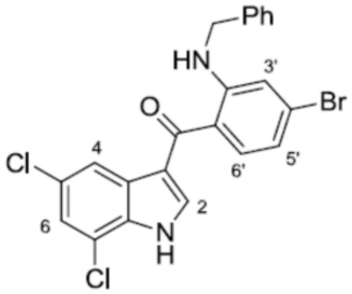

Benzaldehyde (30 mg, 0.286 mmol) was added to a solution of **21** (100 mg, 0.260 mmol) in toluene (2 mL) and the mixture heated at reflux in the presence of 3Å molecular sieves for 6 hours. The mixture was cooled to room temperature before sodium triacetoxyborohydride (83 mg, 0.391 mmol) was added and the reaction stirred for a further 22 hours at room temperature. The solvent was removed under reduced pressure and $NaHCO_3$ (aq.) (5 mL) was added and the product extracted with DCM (3 x 5 mL). The organic extract was dried ($MgSO_4$), filtered and the solvent removed under reduced pressure before the crude product was purified by column chromatography (5-30% EtOAc / Hexanes) giving **9** as an orange-yellow solid (66 mg, 54%). Analytical data for **9** prepared by this method was identical to that obtained when **9** was prepared from **22** (see manuscript).

NMR analysis of the diastereomeric mixtures of **37a/37b**, **38a/38b** and **39a/39b** was challenging. Attempts to assign all ^{1}H NMR spectra have been made and the observed peaks in the ^{13}C spectra have been reported without assignment.

(10b*R*)-5-Benzyl-3-bromo-7,9-dichloro-10b-((2,2-dimethyl-1,3-dioxolan-4-yl)methyl)-5*H*-indolo[2,3-*b*]quinolin-11(10bH)-one 37a/37b

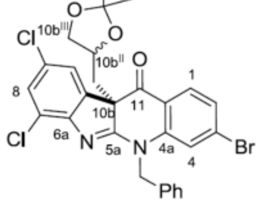

To a solution of **27** (1.00 g, 1.952 mmol) in a mixture of THF (10 mL), EtOAc (10 mL) and water (1 mL) was added NMO (0.46 g, 3.904 mmol) followed by OsO_4 (2.5% in ^{t}BuOH, 1 mL, cat.) and the mixture stirred at room temperature for 18 hours. A saturated solution of Na_2SO_3 (aq.) (25 mL) was added and the mixture was stirred for a further 30 minutes before adding water (25 mL) and EtOAc (50

mL). The mixture was partitioned and the aqueous layer was extracted with EtOAc (3 x 50 mL) before the combined organic extracts were dried ($MgSO_4$), filtered and concentrated to give crude diol (mixture of diastereomers). The crude diol was dissolved in DCM (20 mL) and 2,2-dimethoxypropane (3.05 g, 29.284 mmol) was added followed by PPTS (0.74 g, 2.928 mmol) and the mixture was stirred at room temperature for 18 hours. A saturated solution of $NaHCO_3$ (aq.) (30 mL) was added and the mixture extracted with DCM (3 x 15 mL). The combined organic extracts were dried ($MgSO_4$), filtered and concentrated to give the crude product. Purification by column chromatography (5-20 % EtOAc / Hexanes) gave an inseparable mixture of diastereomers **37a**/**37b** (1:1) as a yellow solid (0.75 g, 65%) (* = **37b**). I.R. (KBr) ν_{max} 3596, 3207, 2921, 1735, 1582, 1553, 1453, 1245, 1093, 1027 cm^{-1}; ^{1}H NMR (500 MHz, $CDCl_3$) δ 7.77 (d, *J* = 8.7 Hz, 1H, C1-H), 7.74 (d, *J* = 8.1 Hz, 1H, C1-H*), 7.67 (d, *J* = 2.0 Hz, 1H, C10-H), 7.62 (d, *J* = 2.0 Hz, 1H, C10-H*), 7.43 – 7.22 (m, 16H, 8 x ArC-H, 8 x ArC-H*), 5.85 – 5.76 (m, 2H, CH_2N, CH_2N*), 5.24 – 5.14 (m, 2H, CH_2N, CH_2N*), 4.06 (ddq, *J* = 11.5, 5.8, 3.0, 2.1 Hz, 1H, C10b^{II}-H), 3.87 (dd, *J* = 8.5, 6.2 Hz, 1H, C10b^{III}-H), 3.62 (p, *J* = 6.1 Hz, 1H, C10b^{II}-H*), 3.39 (dd, *J* = 8.3, 5.8 Hz, 1H, C10b^{III}-H), 3.30 (dd, *J* = 8.4, 5.7 Hz, 1H, C10b^{III}-H*), 3.07 (dd, *J* = 8.4, 7.0 Hz, 1H, C10b^{III}-H*), 2.56 – 2.44 (m, 2H, C10b^{I}-H_2, C10b^{I}- H_2*), 2.24 (dd, *J* = 13.8, 6.5 Hz, 1H, C10b^{I}-H_2*), 1.81 (dd, *J* = 13.8, 3.8 Hz, 1H, C10b^{I}-H_2), 1.26 (s, 3H, CH_3*), 1.21 (s, 3H, CH_3), 1.18 (s, 3H, CH_3*), 1.13 (s, 3H, CH_3); ^{13}C NMR (126 MHz, $CDCl_3$) δ 190.9, 190.9, 172.8, 172.2, 149.4, 149.3, 145.1, 144.5, 135.8, 135.3, 135.3, 135.2, 131.4, 130.6, 130.1, 129.8, 129.6, 129.3, 129.3, 129.2, 129.1, 129.1, 128.1, 128.0, 127.0, 126.9, 126.4, 126.3, 124.2, 124.2, 124.0, 123.7, 119.0, 118.7, 118.5, 117.3, 109.7, 108.8, 71.8, 71.6, 69.4, 69.0, 50.2, 49.9, 45.3, 44.0, 26.7, 26.6, 25.4, 25.2; HR MS [ES$^+$]: *m/z* calcd. for $C_{24}H_{24}{}^{79}Br^{35}Cl_2N_2O_3$ 585.0342, found 585.0339 [M+H]$^+$.

(10b*R*,11*R*)-11-Allyl-5-benzyl-3-bromo-7,9-dichloro-10b-((2,2-dimethyl-1,3-dioxolan-4-yl) methyl)-10b,11-dihydro-5*H*-indolo[2,3-*b*]quinolin-11-ol 38a/38b

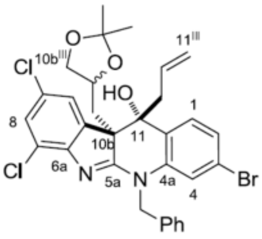

To a solution of **37a**/**37b** (728 mg, 1.242 mmol) in THF (15 mL) at 0 °C was added allylmagnesium chloride (2M in THF, 0.93 mL, 1.863 mmol) and the mixture was stirred at 0 °C for 1 hour. A saturated solution of NH_4Cl (aq.) (25 mL) was added and the organic solvent was removed under reduced pressure before the mixture was extracted with DCM (3 x 20 mL). The combined organic extracts were dried ($MgSO_4$), filtered and concentrated before purification by column chromatography (10-20% EtOAc / Hexanes) to give an inseparable mixture of diastereomers **38a**/**38b** (1:1) as an orange solid (706 mg, 90%). I.R. (KBr) ν_{max} 3374, 2926, 1550, 1484, 1420, 1204, 1064, 850, 702 cm^{-1}; ^{1}H NMR (500 MHz, $CDCl_3$) δ 7.46 – 7.08 (m, 20H, 10 x ArC-H, 10 x ArC-H*), 5.56 – 5.47 (m, 2H, CH_2N, CH_2N*), 5.46 – 5.38 (m, 1H, C11^{II}-H), 5.33 – 5.23 (m, 1H, C11^{II}-H*), 5.22 – 5.15 (m, 2H, CH_2N, CH_2N*), 5.10 – 5.05 (m, 1H, C11^{III}-H_2*), 4.91 (d, *J* = 17.0 Hz, 1H, C11^{III}-H_2*), 4.83 (dt, *J* = 10.3, 2.2 Hz, 1H, C11^{III}-H_2), 4.58 (ddd, *J* = 17.2, 5.8, 1.8 Hz, 1H, C11^{III}-H_2), 4.20 (dtd, *J* = 9.5, 6.2, 1.8 Hz, 1H, C10b^{II}-H/C10b^{II}-H*), 3.97 (dd, *J* = 8.6, 6.3 Hz, 1H, C10b^{III}-H_2/C10b^{III}-H_2*), 3.55 – 3.48 (m, 1H, C10b^{II}-H/C10b^{II}-H*), 3.37 (dd, *J* = 8.6, 6.2 Hz, 1H, C10b^{III}-H_2/C10b^{III}-H_2*), 3.17 – 3.13 (m, 1H, C10b^{III}-H_2/C10b^{III}-H_2*), 2.99 (t, *J* = 7.9 Hz, 1H, C10b^{III}-H_2/C10b^{III}- H_2*), 2.41 – 2.12 (m, 6H, C10b^{I}-H_2, C10b^{I}-H_2*, C11^{I}-H_2, C11^{I}-H_2*), 2.11 – 1.97 (m, 2H, C11^{I}-H_2, C11^{I}- H_2*), 1.36 (s, 3H, CH_3/CH_3*), 1.24 (s, 3H, CH_3/CH_3*), 1.23 (s, 3H, CH_3/CH_3*), 1.14 (s, 3H, CH_3/CH_3*); ^{13}C NMR (126 MHz, $CDCl_3$) δ 175.7, 174.0, 151.7, 151.5, 139.4, 138.9, 138.4, 138.2, 135.9, 135.9, 132.1, 131.0, 129.4, 129.2, 129.1, 129.0, 128.9, 128.9, 128.5, 128.4, 128.3, 128.1, 127.9, 127.7, 127.3, 127.3, 127.2, 126.0, 123.2, 123.1, 122.0, 121.6, 118.8, 118.7, 118.3, 110.7, 108.2, 75.9, 75.4, 72.3, 72.1, 69.9, 69.6, 61.2, 60.5, 49.5, 49.1, 40.7, 40.6, 37.4, 35.9, 26.7, 26.6, 25.6, 25.5; HR MS [ES$^+$]: *m/z* calcd. for $C_{31}H_{30}{}^{79}Br^{35}Cl_2N_2O_3$ 627.0811, found 627.0811 [M+H]$^+$.

(3a*R*,13b*R*)-13b-Allyl-9-benzyl-11-bromo-5,7-dichloro-2,3,9,13b-tetrahydrofuro[3,2-*c*]indolo [2,3-*b*]quinolin-2-ol 39a/39b

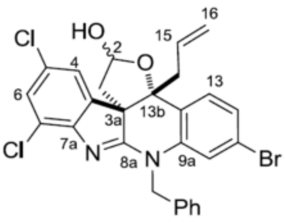

To a solution of **38a**/**38b** (700 mg, 1.114 mmol) in MeOH (35 mL) was added PPTS (308 mg, 1.225 mmol) and the mixture was heated to 60 °C for 5 hours. The solvent was removed under reduced pressure and the mixture was redissolved in DCM (25 mL) before $Pb(OAc)_4$ (741 mg, 1.671 mmol) was added. The mixture was stirred for 1 hour at room temperature before being filtered through a plug of Celite. The plug was washed with DCM (50 mL). The combined organic filtrate was washed with a saturated solution of $NaHCO_3$ (aq.) (30 mL) before drying ($MgSO_4$), filtering and concentrating *in vacuo*. The crude product was purified by column chromatography (5-20% EtOAc / Hexanes) to give an inseparable mixture of diastereomers **39a**/**39b** as a yellow solid (482 mg, 78%). I.R. (KBr) ν_{max}: 3355, 2921, 1553, 1486, 1425, 1204, 1066, 850 cm^{-1}; ^{1}H NMR (500 MHz, $CDCl_3$) δ 7.63 (d, *J* = 2.0 Hz, 1H, C4-H), 7.37 (d, *J* = 8.2 Hz, 1H, C13-H*), 7.28 – 7.16 (m, 13H, 13 x ArC-H/ArC-H*), 7.14 – 7.07 (m, 3H, 3 x ArC-H/ArC-H*), 7.03 – 6.97 (m, 2H, 2 x ArC-H/ArC-H*), 5.79 (t, *J* = 5.9 Hz, 1H, C2-H*), 5.65 – 5.56 (m, 2H, CH_2N, CH_2N*), 5.36 (dd, *J* = 6.7, 1.5 Hz, 1H, C2-H), 5.04 – 4.89 (m, 4H, CH_2N, CH_2N*, C15-H, C15-H*), 4.68 – 4.61 (m, 2H, C16-H_2*), 4.42 – 4.34 (m, 2H, C16-H_2), 2.68 (dd, *J* = 13.8, 6.6 Hz, 1H, C3-H_2), 2.54 (dd, *J* = 13.4, 8.0 Hz, 1H, C14-H_2), 2.46 – 2.38 (m, 4H, 1H for C3-H_2*, 3H for C14-H2/C14-H_2*), 2.34 (dd, *J* = 13.6, 5.9 Hz, 1H, C3-H_2*), 2.01 (dd, *J* = 13.8, 1.5 Hz, 1H, C3-H_2); ^{13}C NMR (126 MHz, $CDCl_3$) δ 172.1, 171.7, 150.4, 150.4, 139.7, 139.2, 135.9, 135.8, 130.3, 129.4, 129.2, 129.2, 129.0, 128.9, 128.9, 128.7, 128.2, 127.8, 127.1, 127.0, 126.5, 126.4, 126.3, 125.8, 123.6, 123.4, 123.1, 123.0, 123.0, 121.0, 120.8, 120.6, 118.2, 118.0, 97.9, 97.2, 86.9, 85.3, 61.8, 59.1, 49.4, 46.2, 45.1, 42.2, 42.0 (C14). HR MS [ES^{+}]: *m*/*z* calcd. for $C_{27}H_{22}{}^{79}Br^{35}Cl_2N_2O_2$ 555.0236, found 555.0233 $[M+H]^{+}$.

X-ray Crystallography data

Table S3. Selected crystallographic data.

	**27**	**41**
empirical formula	$C_{25}H_{17}BrCl_2N_2O$	$C_{27}H_{21}BrCl_2N_2O$
fw	512.23	540.29
crystal description	Yellow prism	Colourless prism
crystal size [mm^3]	0.10×0.10×0.10	0.10×0.10×0.10
space group	$Pna2_1$	$P\overline{1}$
a [Å]	10.199(3)	9.109(4)
b [Å]	21.864(5)	9.470(5)
c [Å]	9.510(2)	14.999(8)
α [°]		94.141(8)
β [°]		97.211(11)
γ [°]		112.194(12)
vol [Å]^3	2120.8(9)	1178.3(10)
Z	4	2
ρ (calc) [g/cm^3]	1.604	1.523
μ [mm^{-1}]	2.217	1.999
F(000)	1032	548
reflections collected	12803	7494
independent reflections (R_{int})	3735 (0.0722)	4157 (0.0637)
parameters, restraints	280, 1	298, 0
GOF on F^2	0.994	1.055
R_1 [$I > 2\sigma(I)$]	0.0422	0.0609
wR_2 (all data)	0.0858	0.1489
Flack parameter	0.043(10)	-
largest diff. peak/hole [e/Å^3]	0.45, -0.46	0.72, -0.67

References

[S1] Yang, X.Y.; Haug, C.; Yang, Y.P.; He, Z.S.; Ye, Y. Stereoselective synthesis of syn- and anti-3a-hydroxyl-1,2,3,3a,8,8a-hexahydropyrrolo[2,3-b]indole-2-carboxylic acid. *Chin. Chem. Lett.* **2003**, *14*, 130–132.

[S2] Li, Y.; Dolphin, D.; Patrick, B.O. Synthesis of a BF_2 complex of indol-2-yl-isoindol-1-ylidene-amine: a fully conjugated azadipyrromethene. *Tetrahedron Lett.* **2010**, 51, 811–814. [doi: 10.1016/j.tetlet.2009.12.003]

[S3] Ohno, M.; Spande, T.F.; Witkop, B. Cyclization of tryptophan and tryptamine derivatives to pyrrolo[2,3-b]indoles. *J. Am. Chem. Soc.* **1968**, *90*, 6521–6522. [doi: 10.1021/ja01025a055]

[S4] Staskun, B. Azepino[1,2-a]indole synthesis from a 1,2,3,4-tetrahydro-9(10H)-acridinone and sodium dichloroisocyanurate or singlet oxygen. *J. Org. Chem.* **1988**, 53, 5287–5291. [doi: 10.1021/jo00257a015]

[S5] Bal, B.S.; Childers, W.E., Jr.; Pinnick, H.W. Oxidation of α,β-unsaturated aldehydes. *Tetrahedron* **1981**, *37*, 2091–2096. [doi: 10.1016/S0040-4020(01)97963-3]

[S6] Kwong, F.Y.; Klapars, A.; Buchwald, S.L. Copper-Catalyzed Coupling of Alkylamines and Aryl Iodides: An Efficient System Even in an Air Atmosphere. *Org. Lett.* **2002**, *4(4)*, 581–584. [doi: 10.1021/ol0171867]

[S7] Feldman, K.S.; Vidulova, D.B. Photochemistry of 2-indolyl aryl ethers: An unexpected rearrangement leading to C-C bond formation. *Heterocycles* **2003**, *60*, 1615-1623. [doi: 10.3987/COM-03-9762]

[S8] Bourdais, J.; Mahieu, C. *Comptes Rendus Des Séances de l'Académie des Sciences, Série C: Sciences chimiques* **1967**, 264(*12)*, 1075-1078.

[S9] Heath-Brown, B.; Philpott, P.G. The indole series. I. Indolylalkylamines. *J. Chem. Soc.* **1965**, 7165-7178 [doi: 10.1039/jr9650007165]

[S10] Voute, N.; Philp, D.; Slawin, A.M.Z.; Westwood, N.J. Studies on the Claisen rearrangements in the indolo[2,3-b]quinoline system. *Org. Biomol. Chem.* **2010**, 8, 442–450. [doi: 10.1039/B915677A]

[S11] Johnston, C.A.; Wilkie, R.P.; Krauss, H.; Neal, A.R.; Slawin, A.M.Z.; Lebl, T.; Westwood, N.J. Polycyclic ethers and an unexpected dearomatisation reaction during studies towards the bioactive alkaloid, perophoramidine. *Tetrahedron* **2018**, *74*, 3339–3347. [doi: 10.1016/j.tet.2018.04.086]

[S12] Arcadi, A.; Bianchi, G.; Marinelli, F. Gold(III)-catalyzed annulation of 2-alkynylanilines: a mild and efficient synthesis of indoles and 3-haloindoles. *Synthesis* **2004**, *4*, 610-618. [doi: 10.1055/s-2004-815947]

[S13] Flatt, A.K.; Yao, Y.; Maya, F.; Tour, J.M. Orthogonally Functionalized Oligomers for Controlled Self-Assembly. *J. Org. Chem.* **2004**, 69, 1752–1755. [doi: 10.1021/jo035821b]
